# Lived experience at the core: A classification system for risk-taking behaviours in bipolar

**DOI:** 10.1177/20552076241269580

**Published:** 2024-08-05

**Authors:** Daisy Harvey, Paul Rayson, Fiona Lobban, Jasper Palmier-Claus, Steven Jones

**Affiliations:** 1 Spectrum Centre for Mental Health Research, Division of Health Research, Faculty of Health and Medicine, Lancaster University, Lancaster, UK; 2 UCREL Research Centre, School of Computing and Communications, InfoLab21, Lancaster University, Lancaster, UK; 3 2099 Lancashire & South Cumbria NHS Foundation Trust, Lancashire, UK

**Keywords:** Bipolar, lived experience, risk-taking, content analysis, corpus linguistics, computational linguistics

## Abstract

**Objective:**

Clinical observations suggest that individuals with a diagnosis of bipolar face difficulties regulating emotions and impairments to their cognitive processing, which can contribute to high-risk behaviours. However, there are few studies which explore the types of risk-taking behaviour that manifest in reality and evidence suggests that there is currently not enough support for the management of these behaviours. This study examined the types of risk-taking behaviours described by people who live with bipolar and their access to support for these behaviours.

**Methods:**

Semi-structured interviews were conducted with *n = *18 participants with a lived experience of bipolar and *n *= 5 healthcare professionals. The interviews comprised open-ended questions and a Likert-item questionnaire. The responses to the interview questions were analysed using content analysis and corpus linguistic methods to develop a classification system of risk-taking behaviours. The Likert-item questionnaire was analysed statistically and insights from the questionnaire were incorporated into the classification system.

**Results:**

Our classification system includes 39 reported risk-taking behaviours which we manually inferred into six domains of risk-taking. Corpus linguistic and qualitative analysis of the interview data demonstrate that people need more support for risk-taking behaviours and that aside from suicide, self-harm and excessive spending, many behaviours are not routinely monitored.

**Conclusion:**

This study shows that people living with bipolar report the need for improved access to psychologically informed care, and that a standardised classification system or risk-taking questionnaire could act as a useful elicitation tool for guiding conversations around risk-taking to ensure that opportunities for intervention are not missed. We have also presented a novel methodological framework which demonstrates the utility of computational linguistic methods for the analysis of health research data.

## Introduction

It is estimated that there are over 1 million people living in the UK with bipolar; ‘a severe mental illness characterised by significant and sometimes extreme changes in mood and energy’.^1^ The present study (unless quoting from literature verbatim) refers to the diagnostic term ‘bipolar disorder’ as ‘bipolar’ throughout. The Lancet Commission^2^ states that terms such as ‘disorder’ which are used in the ICD (International Classification of Diseases)^3^ and the DSM-5 (Diagnostic and Statistical Manual of Mental Disorders, Fifth Edition)^4^ can ‘victimise, criminalise, or misrepresent people with mental health conditions’, and the overwhelming majority of the people with lived experience who were involved in the study preferred to refer to their diagnosis as ‘bipolar’.^2^ Having bipolar increases an individual's risk of suicide by 20 times, and people living with bipolar are estimated to live roughly 10–15 years less than the average population.^5^ The total burden of disease for mental illness in the UK is £117.9 billion a year, and bipolar accounts for roughly 17% of this (£20 billion).^6^

Clinical observations suggest that individuals living with bipolar face difficulties in regulating emotions and impairments to their cognitive processing, which can contribute to high-risk behaviours.^7^ From a diagnostic perspective, the DSM-5^4^ refers indirectly to risk-taking behaviours as part of manic, hypomanic and depressive episodes in the diagnostic criteria for bipolar and related disorders. Risk-taking in mania and hypomania (although not defined explicitly) is described as ‘excessive involvement in activities that have a high potential for painful consequences (e.g. engaging in unrestrained buying sprees, sexual indiscretions, or foolish business investments)’.^4^ The risky behaviours described as part of depressive episodes include suicidal ideation and suicide attempts. Meanwhile, the ICD-10 provides a more ambiguous description of risk-taking, stating that ‘loss of normal social inhibitions may result in behaviour that is reckless, foolhardy, or inappropriate to the circumstances, and out of character’, and does not provide any examples of specific risk-taking behaviours.^3^ Reinharth et al.^7^ succinctly state that ‘it is difficult to measure risk-taking’ in bipolar; an issue which is also reported by Ramirez-Martin et al. in their systematic review of impulsivity, decision-making and risk-taking in bipolar.^8^ Whilst there are a number of tools which can be used to report on risk-taking behaviours such as the DOSPERT (Revised and Improved 30-Item Domain-Specific Risk-Taking Scale^9,10^), the RTI (The Risk-Taking Index^11^) and the ‘dangerous activities’ subscale of the RSDQ (Response Styles to Depression Questionnaire^12–14^), there is currently no bipolar-specific validated measure. In response to the lack of a validated tool, Reinharth et al.^7^ tested a self-report questionnaire based on three pre-existing measures (the ‘dangerous activities’ subscale of the RSDQ (Response Styles to Depression Questionnaire^12–14^), the Multi Impulsivity Scale (MIS)^15^ and the Clinical Assessment of Multi-impulsivity (CAM) checklist).^16^ The authors^7^ used an adapted version of this questionnaire to observe risk-taking behaviours in a sample of people with a diagnosis of bipolar to demonstrate that ‘self-damaging risk-taking behavior is a regular occurrence for patients with bipolar spectrum disorders’, recommending that a validated measurement tool for risk-taking behaviours in bipolar should be developed.^7^

The majority of existing research which focuses on risk-taking in bipolar relies on laboratory tasks or behavioural experiments to measure risk-taking propensity or focus on the cognitive mechanisms which may contribute to risk-taking,^17–23^ whilst a much narrower range of papers focus specifically on the types of risk-taking behaviours that present in bipolar, which is the focus of the present study. These studies are generally limited by reporting only on the behaviours associated with specific mood states such as hypomania^24^ or by relying solely upon pre-existing questionnaires; which restrict participants from talking about behaviours that are not included in these measurement tools.^7^ Thus, the aim of the current study was to develop a representative classification of risk-taking behaviours based on real-life experiences across different mood states, using data generated from interviews with people with lived experience (PWLE) and healthcare professionals (HPs). The analyses performed throughout this study unite to provide a conceptual classification system of risk-taking behaviours in bipolar and evidence that further support is needed to help manage these behaviours. This support may include more targeted interventions for risk-taking behaviours such as developing specific care plans which focus on preventing or minimising risk-taking behaviours or improving access to psychologically informed care. We also provide evidence which suggests that the use of a standardised risk-taking questionnaire could help to de-stigmatise conversations around risk-taking by normalising these behaviours, improve awareness of the types of behaviours that are relevant to talk about, and also make it easier to talk about typically ‘taboo’ topics that people with a diagnosis of bipolar may not otherwise feel that they can address.

This study presents a unique window into real-life experiences of risk-taking, implementing a novel research methodology for the study of risk-taking behaviours (to the best of our knowledge). By incorporating corpus linguistics^a^ into our study design, we have been able to identify not only the risk-taking behaviours which are tangible within our data, but we have also been able to observe how participants talk about this topic and patterns that emerge from the textual data. As described by Tausczik and Pennebaker, applying computational linguistic methods to language can provide a window into emotional and cognitive worlds.^25^ Recent studies demonstrate the importance of a methodology which takes a ‘lived-experience-centric’ approach^26–28^ and there are a growing number of studies which report interesting findings by combining computational linguistic methods with lived experience interviews within the domain of mental health research.^29–33^

The main objectives of this study were:

1. To identify the risk-taking behaviours described by participants (PWLE and HPs) and develop a classification of these behaviours using content analysis based primarily on lived experience.
2. To compare the risk-taking behaviours described by interview participants with behaviours from a risk-taking questionnaire to understand the relevance of a risk-taking measurement tool in clinical practice.
3. To identify areas where further support is needed for managing risky behaviours as well as issues associated with stigmatisation using corpus methods and qualitative analysis.

## Methods

This study presents a novel mixed-method multi-method exploration into the lived experience of risk-taking behaviours in bipolar, integrating two primary sources of data (1) textual data from interview transcripts and (2) numerical data from a risk-taking questionnaire. Both qualitative and quantitative methods were adopted through content analysis and corpus linguistic analysis of the textual interview data, and quantitative methods were further utilised through statistical analysis of the risk-taking questionnaire. The methodological elements which comprise our framework were selected based on our pragmatist ontological stance, i.e. using the philosophical and/or methodological approaches that work best for the particular research problem that is being investigated.^34^ This novel framework has thus, by definition, not been utilised in existing research, although successes of individual methodological elements have been referenced in prior studies, including the use of Questerviews (integrating questionnaires within qualitative interviews),^35–39^ content analysis to develop the initial coding toolkit for the risk-taking behaviours^40–42^ and corpus linguistic methods for analysis of the interview data.^29,43,44^

The COREQ checklist for this research can be found in Supplementary Material S2.^45^

### Recruitment

Two groups of participants were recruited for the interviews between May and November 2022: (1) participants with a self-reported diagnosis of bipolar who were 18 or older, and (2) HPs with at least 5 years’ experience working with individuals with a diagnosis of bipolar. PWLE participants were recruited using purposive sampling via the ‘People in Research’ website^46^ and through Lancaster University's Spectrum Connect research network. The HPs were recruited either via the ‘People in Research’ website^46^ or directly by email (in the case of academic psychiatrists, psychologists and GPs with an expertise in bipolar). The HPs who were directly contacted by email were identified through the research team's network or via an online search for HPs with expertise in bipolar. The primary focus of the interviews was to collect lived experience narratives, but the insight from a small number of HPs who were familiar with the heterogeneity of the diagnosis was considered to be useful in providing a broader understanding of risk-taking behaviours and how these are observed from both a lived experience and healthcare perspective. The target number of participants was *n = *15 PWLE and *n = *5 HPs, with the decision to interview a relatively small number of participants informed by the principle of sufficiency.^47^

All participants who expressed an interest in participating in the study were first sent an information sheet, asked to provide some basic personal details and sign an informed consent document (the participant information sheets can be found in 1.1 and 1.2 of the Supplementary Material S1 document). PWLE participants were asked to self-report their diagnosis and for how long they have lived with the diagnosis, as well as provide the contact details of their healthcare team which could be used in case of emergency. In the two weeks prior to the interview being conducted, participants with a diagnosis were asked to take part in a short screening call to confirm a euthymic mood state, for which the Mood Disorder Questionnaire (MDQ),^48^ the Altman mania scale^49^ and the 16-Item Quick Inventory of Depressive Symptomatology Self-Report (QIDS-SR) were used.^50^^b^ If participants scored within the required limits on the screening questionnaires, they were invited to interview within the following two weeks. Those participants who did not score within the accepted range were invited to take the screening tests again after two weeks.

### The interview process

The interviews took place remotely via Microsoft Teams or by telephone and were either recorded directly through Microsoft Teams or through an encrypted device (in accordance with the ethics approval granted to this project by Lancaster University), and all recordings were converted to M4A format. The audio recordings were then imported to NVivo transcription software^51^ which processed the audio and attempted to automatically convert the speech to text. This process increased the efficiency and speed of the overall transcription process but the resulting text after automatic transcription still included errors. The transcripts were edited manually to correct errors and to remove hesitations, non-verbal utterances (e.g. ‘uh’ and ‘er’) and repetition, resulting in a naturalised form of transcription (deemed acceptable for this task because the analysis is semantic not phonetic).^52^ The transcripts were also anonymised at this stage, with the following masking techniques being incorporated:

- All names and other specific identifying information was removed where possible or replaced with placeholder text where removing the entity would affect comprehension of the text, e.g. ‘Sam’ could be converted to ‘my husband’.
- Specific place names were replaced with county names, e.g. Wimbledon would be converted to London.
- References to specific hospitals and trusts were removed.

The transcripts were then converted to separate XML files and were annotated using XML markup.

The interviews involved two parts (1) a semi-structured interview centred around open-ended questions and (2) a Likert-item risk-taking questionnaire based on the modified MIS-CAM risk-taking survey proposed by Reinharth et al.^7^ which was an adaptation of two surveys (the MIS and the CAM) originally produced by Lacey and Evans.^15^ The MIS is a Multi Impulsivity Scale which records 22 items related to impulsivity in a self-report questionnaire, and the CAM is the Clinical Assessment of Multi-Impulsivity – it measures the same behavioural domains as the MIS but allows clinicians to add extra detail.^16^^c,d^ The risk-taking questionnaire used in this research has not been validated and was not used to measure risk-taking behaviours or impulsivity, rather it was used as a triangulation tool to identify differences between the behaviours that participants chose to disclose during the open-ended interview questions and those that they identified within the questionnaire. It also acted as an elicitation tool within the interviews to ask participants how they felt about completing such a questionnaire and whether they believed a similar tool would be useful in clinical practice. The inclusion of this questionnaire enabled us to assess whether the risky behaviours which are included in this survey were deemed relevant by the interview participants or whether participants reported additional risk-taking behaviours which would be useful to include in any future measurement tool, as previous work has acknowledged the absence of a bipolar-specific measure of risk-taking behaviours and the importance of developing validated tools in the future.^7^

In the majority of cases participants completed the risk-taking questionnaire during the interview after the open-ended interview questions, but in some cases the questionnaire was completed independently by the participant prior to the interview due to accessibility needs, for example, where participants requested a telephone call instead of a video call and preferred to complete the questionnaire ahead of the interview taking place. The interviews were all conducted by one female researcher (DH) as part of their PhD thesis, and all interviews took place in a private location. Prior to conducting the interviews, the interviewer took part in internal training provided by Lancaster University for qualitative interviews as well as additional training provided by the Social Research Association in ‘Managing Challenging Interviews’.

The open-ended interview questions were centred around four main themes as defined in the interview schedules: (1) defining risk-taking and personal experiences of risk-taking, (2) the impact of risk-taking behaviours, (3) feelings and emotions related to risk-taking and (4) access to support. The full interview schedules can be found in Supplementary Material S1 (1.3 and 1.4). The interview schedule and adapted questionnaire were pilot tested internally within the research team and additionally with one member of Lancaster University's Spectrum Connect Advisory Panel.

All participants were offered a £30 reimbursement for taking part in the interview which was either paid directly to the participant or donated to a charity of their choice at their request. The final transcripts were anonymised to mask names, places and other identifying information. No repeat interviews were conducted.

### Analysis framework for the study

**Figure 1.**
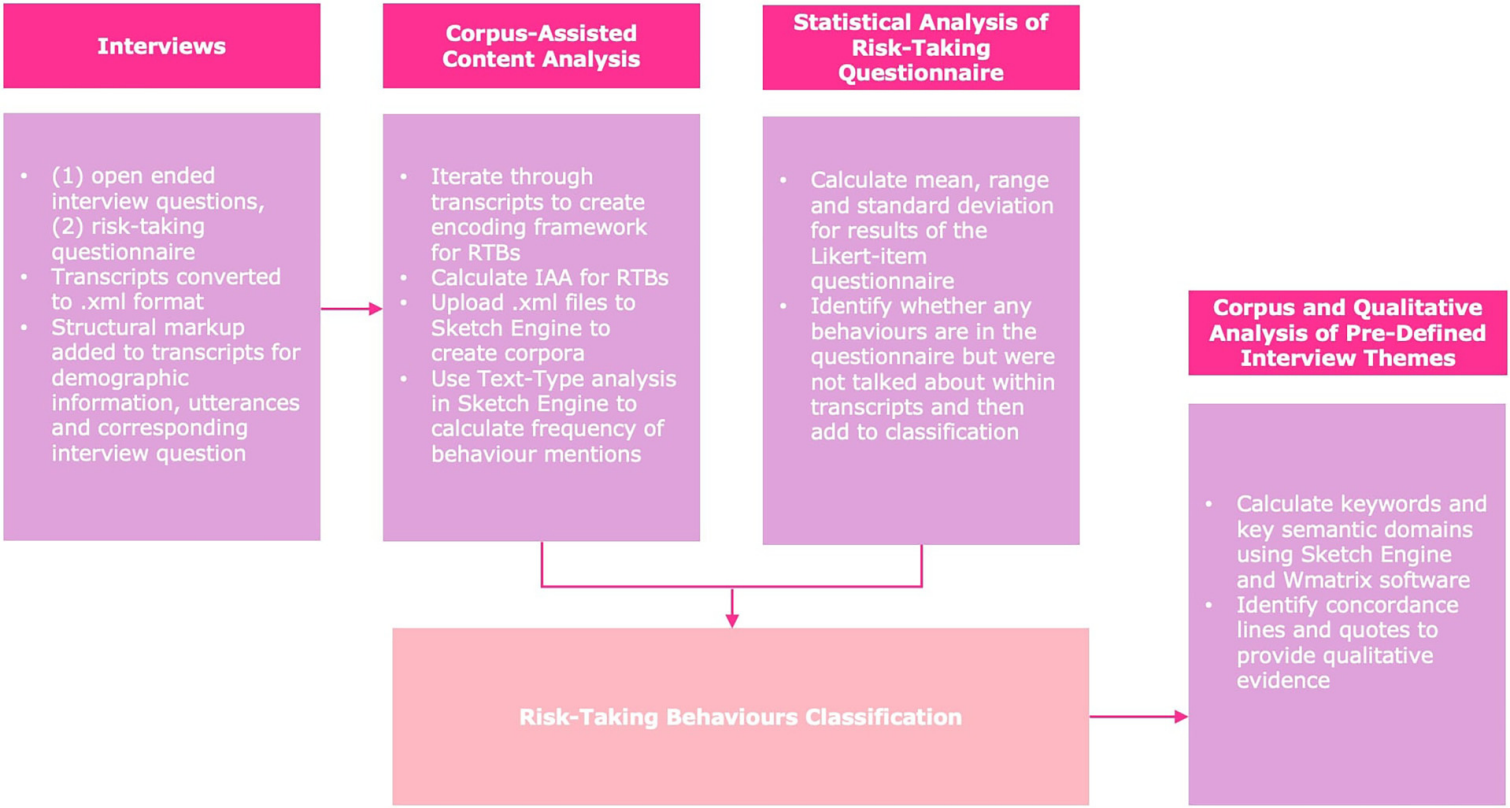
Diagram of the analysis framework. RTB: risk-taking behaviour; IAA: Inter-Annotator Agreement.

This study integrated two primary sources of data (1) textual data from interview transcripts and (2) statistical data from a risk-taking measure, with both sets of data generated during the interviews. The analysis framework for this data included three stages. First, content analysis was conducted on the interview transcripts to encode risk-taking behaviours described by participants and create a classification system. Second, we performed statistical analysis of a risk-taking questionnaire^7^ which was completed by the same interview participants as part of the interview process, and finally we used corpus analysis to identify how risk-taking behaviours were talked about in relation to the four key themes which formed the structure of the interview schedule (see ‘The interview process’). Figure 1 demonstrates the analysis framework for this study.

#### Content analysis

The first part of the analysis for this study was to produce the encoding for the risk-taking behaviours using content analysis, which is the process of compressing text into ‘content categories based on explicit rules of coding’.^53^

We employed a grounded theory approach to content analysis^54^ which enabled the researchers to develop a coding framework as the coding progressed, based on the data within the transcripts (emergent coding) which was then used as a rubric to re-code existing data and encode future data. We integrated the content analysis within the corpus building process by annotating the transcripts using XML markup so that the encoded behaviours could later be interpreted by the corpus software Sketch Engine.^55^ The encoding of risk-taking behaviours was performed primarily by DH, and a second annotator (PR) independently annotated 40% of the transcripts to calculate Inter Annotator Agreement (IAA). Disagreements were discussed within the research team (SJ, PR, FL, DH) resulting in the final coding framework which was applied to all transcripts.

IAA was calculated using an *F*-measure^56^ for two conditions; span matches (both annotators annotated a text span with a risk-taking behaviour) and concept matches (both annotators annotated a text span with the *same* risk-taking behaviour^57^). A ‘relaxed’ approach to matches was implemented as described in Wang et al.^57^ In this method, both annotators did not need to mark the exact same span of text. Instead, agreement was considered valid if their annotations overlapped within any part of the text span. The weighted F1 IAA scores for span matching and concept matching, respectively, were 0.79 and 0.54. Disagreements between annotators regarding the risk-taking tags and tags which achieved a low F1 agreement score were discussed with the wider research team (SJ, PR, FL, DH) until agreement was met, and this process resulted in the iterative emergent coding process until the final list of risk-taking behaviours presented in the classification system in section ‘Suggested classification system for risk-taking behaviours’ (and extended in Supplementary Material S1 (2.1, Table S1) was produced.

After the encoding had been finalised, the transcripts were imported into Sketch Engine^55^ which is a corpus analysis software capable of interpreting XML markup, thus enabling us to interpret our annotations and view representations graphically. A separate corpus was generated for each group (the PWLE corpus was 92,105 words and the HP corpus was 18,335 words.)

The classification system presents quantitative evidence demonstrating the frequency of the risk-taking behaviours encoded in the transcripts (see section ‘Suggested classification system for risk-taking behaviours’), which was calculated using the Text Type tool within Sketch Engine.^55^ Using this tool we were able to observe each of the risk-taking behaviours we had identified and annotated within the context of the whole corpus. The framework for content analysis that we employed in this study featured both qualitative and quantitative elements; we explored the transcripts qualitatively to identify the risk-taking behaviours and how these were presented contextually, and we then used quantitative analysis within Sketch Engine^55^ to produce the classification system based on frequency and distribution across participants.

#### Statistical analysis of the MIS-CAM risk-taking questionnaire

The results from the risk-taking survey were analysed statistically using (1) Cronbach's alpha^58^ (to determine whether the items within the survey are related to a consistent construct by measuring the level of variance between individual responses), (2) mean, (3) range and (4) standard deviation, and have also been presented graphically in diverging bar graphs to demonstrate the distribution of responses across participants (section ‘The risk-taking questionnaire (modified MIS-CAM)’). Both groups completed the questionnaire; with the PWLE group answering the questions based on their personal experiences and the HP group answering the questions based on their clinical observations. The results of the questionnaire were compared to the behaviours encoded during content analysis and any additional behaviours were incorporated into our classification of risk-taking behaviours.

#### Corpus analysis

After generating the classification system, we used Sketch Engine^55^ and Wmatrix^59^ for the final stage of corpus analysis. We performed keyword and concordance analysis with Sketch Engine whilst Wmatrix enabled us to analyse the key semantic domains represented in our corpora. Only the utterances from participants were included across all of the analyses (i.e. the interviewer utterances were removed), and the BNC 2014 spoken corpus^60^ (the British National Corpus 2014 spoken part) was used as a reference corpus for the Sketch Engine analysis. The BNC 2014 was chosen because it is a (relatively) recent corpus of over 11 million words which is comparable owing to the spoken data mode.^61,62^ In the Wmatrix analysis, the PWLE corpus served as the focus corpus and the HP corpus acted as the reference corpus so we could directly compare the semantic domains for each group.

This corpus analysis was driven by the risk-taking behaviours identified using the classification system as well as the four key themes of the interviews which were defined at study design (see section ‘The interview process’), enabling us to study more in depth what participants talked about and how they talked about it. Corpus methods were identified as being relevant for the analysis of this data due to the ability to observe high-level and aggregate insights from the text whilst also being able to perform fine-grained analysis on relevant text samples to reveal singularities. The majority of corpus methods are based on the use of statistics to allow the comparison of frequencies, and can be used to analyse not only the words within the corpus, but also the annotations that are applied to these words, i.e. enabling us to observe patterns in usage across the risk-taking behaviour annotations.

As well as annotating the risk-taking behaviours during content analysis, the transcripts were also annotated with structural elements at their inception, including speaker, interview theme and interview question, and the Text Type tool was used within Sketch Engine to generate sub-corpora based on these pre-coded elements. Keywords were used to demonstrate the words which were salient within our focus corpus (either the PWLE or the HP corpus) compared to the reference corpus, where keywords can provide ‘information about the keyness or specificity of a given corpus in terms of what it is about’.^63^ In Sketch Engine, keyness is calculated using the simple maths method, which compares the relative frequencies of the words in both the focus and reference corpus to produce a keyness score. This score essentially translates as ‘*the keyword is X times more frequent in corpus A than corpus B*’.^64^ We defined the parameters within Sketch Engine so that a word had to appear at least three times to be included in the keyword list, and the rare/common focus score was set to 1 so that the preference was for less common words to be included.

Next, we used the USAS (UCREL semantic analysis system) semantic tagger within Wmatrix^59^ to identify overused and underused semantic domains by comparing the corpora from our two groups; thus identifying which sets of semantic items were talked about more or less by each group. When using this tool we only considered items which were statistically significant at the level of *p* < 0.05, which equates to a log-likelihood cut-off value of 3.83.

Finally, after identifying the keywords and key semantic domains using quantitative methods, we identified relevant qualitative examples to use as evidence from concordance lines, which are keywords and phrases located within their surrounding context to the left and right of the search term (KWIC or Key Word In Context). We also manually analysed text within our corpora to make quantitative observations where relevant, for example, to report on ‘*the number of participants who talked about X*’. All quotes and concordance lines that are provided in the results section have been paraphrased and anonymised to protect the participants’ privacy.

### Ethics

Ethics approval was granted for the project by Lancaster University in December 2021 (FHMREC21042). All personal data collected from the interviews has been made anonymous using pseudonyms or an identification number. All references to information that could be used to identify the participant such as places, dates and names have been masked to protect the identity of the speaker. A service user advisory panel at Lancaster University were consulted regarding the design and intent of this research study and their feedback was incorporated into the methodology, for example, one service user recommended including a question about positive risk-taking as well as looking at the negative impacts of risk-taking behaviours.

## Results

### Participant demographics

**Figure 2.**
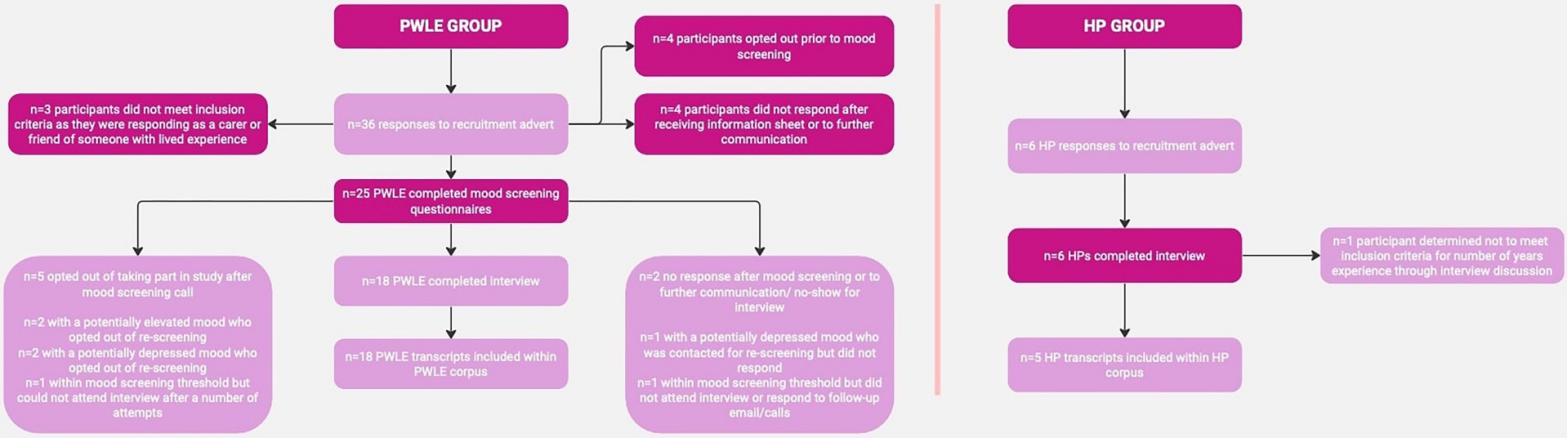
Flowchart illustrating the recruitment process. PWLE: people with lived experience; HP: healthcare professional.

Figure 2 represents the recruitment process, illustrating that 36 PWLE responded to the recruitment advert, of whom 33 met the inclusion criteria and 18 went on to interview. Six HPs responded to the recruitment advert, five of whom met the inclusion criteria. The demographic information for the interview participants is shown in Table 1, and the mean age of diagnosis of bipolar for the PWLE group was 33 years of age. This is consistent with reported data that suggests that the mean age of onset of bipolar is 25^65^ and that it takes an average of 9 years to receive a diagnosis of bipolar from the onset of symptoms.^66^ The interviews ranged from 20 to 80 min and the average interview length was 45 min.

**Table 1.** Participant demographics.

PWLE group demographics			
Variables	Categories	*n* (%)	
**Gender**			
	Female	14 (77)	
	Male	4 (23)	
**Age at diagnosis**			
	16–24	1 (6)	
	25–34	11 (61)	
	35–44	5 (27)	
	45–54	1 (6)	
**Age at time of interview**			
	25–34	1 (6)	
	35–44	6 (34)	
	45–54	5 (27)	
	55–64	1 (6)	
	65+	5 (27)	
**Number of years living with a diagnosis of bipolar**	**Mean**	**Range**	**SD**
	18.9	40	12.7

**HP group demographics**			
**Variables**	**Categories**	***n* (%)**	
**Gender**			
	Female	1 (20)	
	Male	4 (80)	
**Age**			
	25–34	1 (20)	
	35–44	1 (20)	
	45–54	1 (20)	
	55–64	2 (40)	
**Profession**			
	Bibliotherapist	1 (20)	
	Social worker	1 (20)	
	GP and researcher	1 (20)	
	Clinical psychologist	1 (20)	
	Consultant psychiatrist	1 (20)	

### Suggested classification system for risk-taking behaviours

Table 2 presents an abridged version of the classification system for 39 risk-taking behaviours. A more detailed classification table is provided in Supplementary Material S1 (2.1, Table S1); reporting statistics for each risk-taking behaviour; how often these risk-taking behaviours were described by the two groups of participants, how many PWLE participants engaged with each behaviour (and overall domain of behaviour) through their narratives, as well as the mean, range and standard deviation for each behaviour and domain.

**Table 2.** A classification of risk-taking behaviours using participant data from qualitative interviews and the risk-taking questionnaire.

**Risk-taking domain**	**Risk-taking behaviours**
**(Anti-)Social behaviours**	
	‘Having no filter’ (being rude/inappropriate/lack of social boundaries)
	Starting arguments
	Neglecting relationships
	Hyperfixation
	Being aggressive, violent or destructive
	Talking to strangers
**Financial behaviours**	
	Spending money impulsively or excessively
	High-risk investing
	Gambling
	Quitting a job impulsively
	Excessive generosity (giving money/belongings away)
**Dangerous and disinhibited behaviours**	
	Going to dangerous or unusual places
	Leaving home or running away
	Standing on bridges/high places
	Impulsive travelling
	Extreme sports
	Dangerous driving
	Stealing
	Carrying or using weapons
	Wearing inappropriate clothing
	Entering vulnerable relationships
	Walking in traffic
	Fire-starting
**Health-risk and substance-misuse behaviours**	
	Medication non-adherence
	Excessive eating
	Not eating
	Smoking
	Drinking alcohol excessively
	Taking recreational drugs
**Self-injurious and suicidal behaviours**	
	Suicide or attempting suicide
	Suicidal ideation
	Overdosing
	Self-harming
**Sexual behaviours**	
	Having sex with strangers
	Having unprotected sex
	Having an affair
	Porn addiction
	Hypersexuality (dating apps)
	Hypersexuality (non-specific)

We identified 39 risk-taking behaviours based on the content analysis and the results of the risk-taking questionnaire, and we manually inferred six domains from these behaviours to group them; cross-referencing with the domains that have previously been identified in the literature (see Table S3 in Supplementary Material S1 (2.4)); (1) (anti-)social behaviours, (2) financial behaviours, (3) dangerous and disinhibited behaviours, (4) health-risk and substance-misuse behaviours, (5) self-injurious and suicidal behaviours and (6) sexual behaviours.

Analysis of Supplementary Material S1 (2.1, Table S1) demonstrates that the risk-taking behaviours which were most talked about by PWLE participants (based on the behaviours which were cited by the highest number of participants) included spending money excessively, going to dangerous and unusual places and suicidal ideation. There were four behaviours which were described by PWLE but were not talked about by HPs; extreme sports, entering vulnerable relationships, walking in traffic and smoking. There is substantial overlap between the behaviours which were most frequently described by the HP group and the PWLE group, although the behaviour of attempted suicide is overrepresented by the HP group compared to the PWLE group. There were three behaviours which were described by the HP group but not by PWLE, which were: quitting a job impulsively, carrying or using weapons and porn addiction. Having no filter or breaking social boundaries, excessive generosity and leaving home or running away were included within the top 15 behaviours described by the PWLE group but were not included within the most frequent behaviours for the HP group, and gambling, attempting suicide and hyperfixation were included within the top 15 behaviours for the HP group but not the PWLE group. Additional analyses of the behaviour mentions are provided in Supplementary Material S1 (2.2, Figures S1–S4), to demonstrate how the breakdown of behaviour mentions by interview section, i.e. the behaviours described narratively during the open-ended interview questions and those described narratively during the risk-taking questionnaire exercise.

#### Data saturation

Our target number of interviews was 20 (15 PWLE and 5 HP), based on the principle of sufficiency described by Young and Casey,^47^ and the final number of participants we interviewed was 23 (18 PWLE and 5 HP). Although there is little guidance on how to measure data saturation from interview data, Guest et al.^67^ suggest that data saturation is ‘the point in data collection and analysis when new information produces little or no change to the codebook’. Within our study, 90% (35/39) of the encoded behaviours were identified by the 15th interview, with four additional risk-taking behaviours described during the final two interviews with PWLE and the five interviews with the HP group. Based on the data we had collected and the number of repeating themes in the data, we did not extend the recruitment period or conduct more interviews. This aligns with the findings from Young and Casey^47^ who report that ‘rigorously collected qualitative data from small samples can substantially represent the full dimensionality of people's experiences, with larger sample sizes adding important but perhaps increasingly minute pieces of meaning’.

### The risk-taking questionnaire (modified MIS-CAM)

The modified MIS-CAM survey was presented by Reinharth et al.^7^ as a suggested measurement tool for risk-taking behaviours in PWLE of bipolar. The survey was adapted into a Likert-item questionnaire within this study and distributed using Qualtrics software.^68^ The scoring system for the questionnaire was as follows:

- 1: ‘I never do this/have never done this’ (PWLE group) or ‘I have never observed this behaviour’ (HP group);
- 2: ‘I rarely do/have done this’ (PWLE group) or ‘I have rarely observed this behaviour’ (HP group);
- 3: ‘I sometimes do/have done this’ (PWLE group) or ‘I have sometimes observed this behaviour’ (HP group);
- 4: ‘I do/have done this fairly often’ (PWLE group) or ‘I have observed this behaviour fairly often’ (HP group);
- 5: ‘I do/have done this behaviour very frequently’ (PWLE group) or ‘I have observed this behaviour very frequently’ (HP group).

The survey achieved a Cronbach's alpha^58^ of 0.79 for the PWLE group and 0.88 for the HP group which demonstrates high to very high internal consistency for the items within the survey.^69^ The mean, range and standard deviation for each group for all Likert-item questions is shown in Table 3; the behaviours have been ranked from highest to lowest scoring for each group. The standard deviation and range scores for the PWLE group were generally much higher than the HP group, illustrating a higher variance in responses.

**Table 3.** Statistical measures for the risk-taking questionnaire showing the ranked responses for each group.

PWLE group responses				HP group responses			
Risk-taking behaviour	PWLE mean	PWLE SD	PWLE range	Risk-taking behaviour	HP mean	HP SD	HP range
**Suicidal ideation**	3.06	1.16	4	**Recreational drug use**	4.20	0.45	1
**Eat excessive amounts of food**	3.00	1.28	4	**Shop without worrying about running up debts**	4.20	0.84	2
**Shop without worrying about running up debts**	2.94	1.55	4	**Suicidal ideation**	4.00	0.71	2
**Start an argument**	2.69	0.82	3	**Excessive alcohol consumption**	3.80	0.45	1
**Initiate relationships with strangers**	2.56	1.34	4	**Go to a potentially dangerous place where client wouldn’t normally go**	3.60	0.89	2
**Excessive alcohol consumption**	2.39	1.34	4	**Self-harm**	3.20	0.45	1
**Seek out and engage in casual sexual relations**	2.33	1.37	4	**Attempt suicide**	3.00	1.41	3
**Go to a potentially dangerous place where client wouldn’t normally go**	2.28	1.07	3	**Gamble**	3.00	1.00	2
**Dangerous or aggressive driving**	2.28	1.60	4	**Start an argument**	3.00	1.41	3
**Self-harm**	2.11	1.18	4	**Initiate relationships with strangers**	2.80	0.84	2
**Hit someone or break something**	1.94	0.73	2	**Eat excessive amounts of food**	2.60	0.89	2
**Attempt suicide**	1.88	0.89	3	**Hit someone or break something**	2.20	0.84	2
**Gamble**	1.61	0.78	2	**Seek out and engage in casual sexual relations**	2.20	1.30	3
**Recreational drug use**	1.56	1.04	3	**Dangerous or aggressive driving**	2.00	1.00	2
**Make risky investments**	1.56	0.92	3	**Shoplift or steal something**	2.00	0.71	2
**Shoplift or steal something**	1.22	0.73	3	**Make risky investments**	1.80	1.30	3
**Set fire to something**	1.20	0.51	2	**Set fire to something**	1.40	0.55	1

PWLE: people with lived experience; HP: healthcare professional.

For each variable, missing values are replaced with the variable mean.

#### How do the results of the questionnaire differ between the groups?

Table 3 demonstrates that the five most frequent risk-taking behaviours based on the mean scores of the risk-taking questionnaire in the PWLE group were suicidal ideation, eat excessive amounts of food, shop without worrying up debts, start arguments and try to initiate relationships with strangers. For the HP group, the five most frequent behaviours were recreational drug use, shop without worrying about running up debts, suicidal ideation, excessive alcohol consumption and go to a potentially dangerous place where the client wouldn’t normally go. Based on the mean scores of the Likert-item responses, the results show some correlation with the results reported by Reinharth et al.,^7^ who suggested that the behaviours of binge eating, excessive drinking and risky spending were more prevalent in people with a diagnosis of bipolar than a control population, together with gambling and self-injury. Shopping without worrying about running up debts and suicidal ideation scored highly for both groups, and conversely setting fire to something and shoplifting scored low across both groups.

However, there are also some notable differences between the results reported by both groups. Firstly, recreational drug use was one of the highest scoring behaviours for the HP group, whilst it was the fourth most infrequent behaviour for the PWLE participants. There are also a number of behaviours that rank higher for the PWLE participants compared to the HP group such as engaging in casual sexual relations, dangerous driving and eating excessive amounts of food.

**Figure 3.**
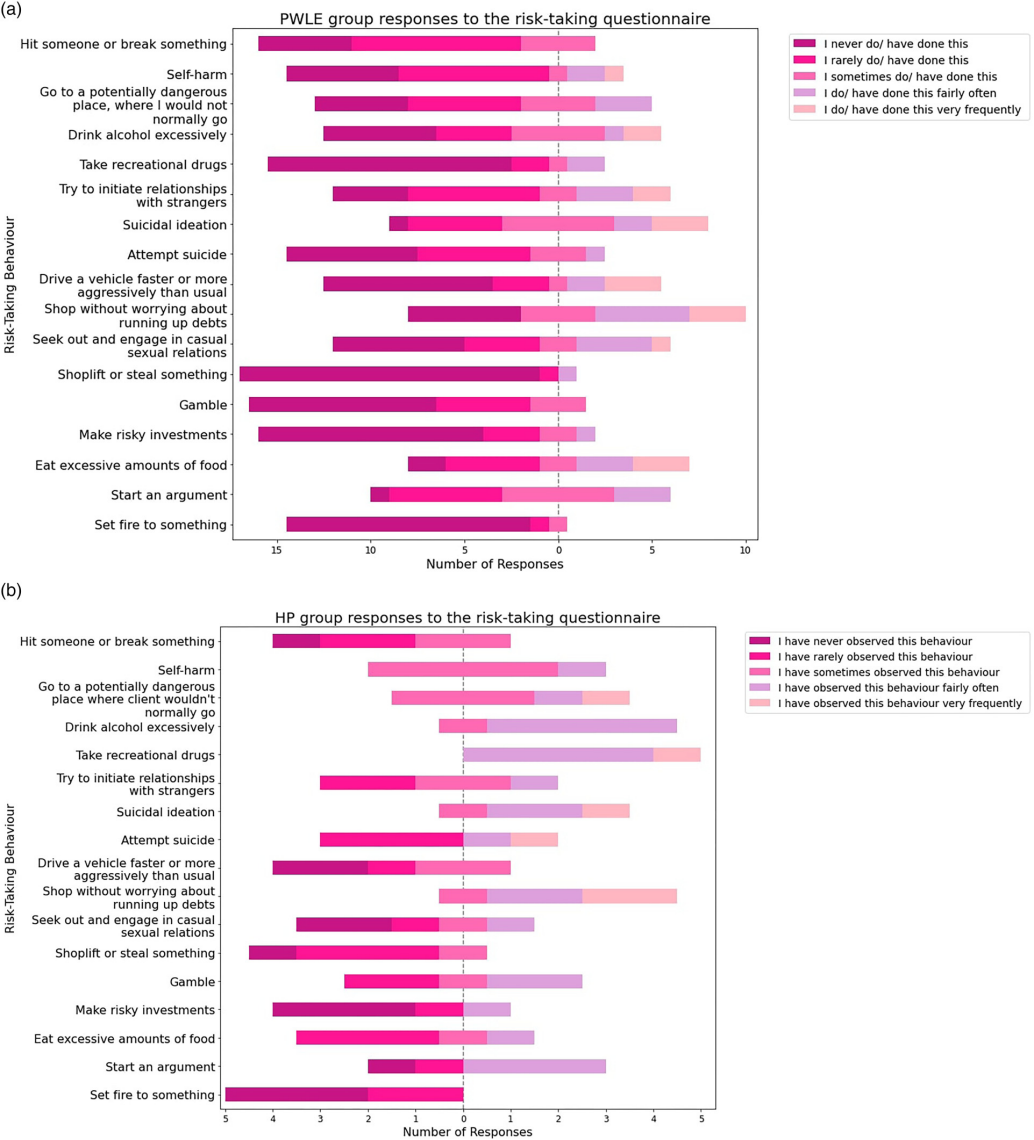
(a) PWLE group responses to the risk-taking questionnaire. (b) HP group responses to the risk-taking questionnaire. PWLE: people with lived experience; HP: healthcare professional.

The distribution of responses for both groups is also represented in the stacked bar charts in Figure 3.

### Corpus analysis and qualitative evidence for key interview themes

The following section presents corpus analysis and qualitative evidence which has been grouped narratively using the key themes that were defined in the interview schedule: (1) defining risk-taking and personal experiences of risk-taking, (2) the impact of risk-taking behaviours, (3) feelings and emotions related to risk-taking and (4) access to support.

#### Defining risk-taking, risky behaviours and personal experiences of risk-taking

Table 4 demonstrates the top 10 keywords for each group using the whole corpus from each group. The keywords for the PWLE group indicate that risk-taking is associated with being ‘unwell’, having manic or hypomanic symptoms and is also talked about in relation to the anti-psychotic drug ‘olanzapine’. The HP group keywords include more references to impulsivity, as well as references to self-harm and [suicidal] ideation.

**Table 4.** Top 10 keywords from PWLE and HP corpora showing how risk-taking is conceptualised.

**Item**	**Frequency (focus)**	**Frequency (reference)**	**Relative frequency (focus)**	**Relative frequency (reference)**	**DOCF (focus)**	**DOCF (reference)**	**Score**
**PWLE group**							
***bipolar***	206	13	1885.5835	1.09863	18	6	898.96
***mania***	36	3	329.51944	0.25353	12	3	263.671
***hypomanic***	25	0	228.83295	0	6	0	229.833
***psychosis***	27	2	247.13959	0.16902	8	2	212.263
***risky***	132	57	1208.23804	4.81706	18	37	207.878
***psychiatrist***	66	25	604.11902	2.11275	15	14	194.4
***olanzapine***	20	0	183.06636	0	6	0	184.066
***unwell***	44	20	402.746	1.6902	10	15	150.08
***disorder***	62	34	567.50574	2.87334	14	23	146.774
***diagnosis***	40	19	366.13272	1.60569	12	15	140.897
**HP group**							
**Item**	**Frequency (focus)**	**Frequency (reference)**	**Relative frequency (focus)**	**Relative frequency (reference)**	**DOCF (focus)**	**DOCF (reference)**	**Score**
***bipolar***	66	13	3062.64502	1.09863	5	6	1459.832
***impulsive***	20	2	928.07422	0.16902	3	2	794.746
***mania***	16	3	742.45941	0.25353	2	3	593.093
***impulsivity***	12	0	556.84454	0	1	0	557.845
***self-harm***	12	1	556.84454	0.08451	5	1	514.375
***ideation***	10	0	464.03711	0	4	0	465.037
***misuse***	15	8	696.05566	0.67608	2	6	415.885
***relapse***	15	8	696.05566	0.67608	4	6	415.885
***dbt***	8	0	371.22971	0	1	0	372.23
***risky***	46	57	2134.5708	4.81706	5	37	367.122

PWLE: people with lived experience; HP: healthcare professional; DOCF: document frequency.

PWLE overwhelmingly described that risk-taking behaviours were related specifically to their mood states (*n = *17/18, 94%). Participants described that different risks were associated with a high or a low episode, and a number of participants stated that risk-taking was related to the impulsivity that comes with hypomania or mania. Four (22%) participants described that it could sometimes be difficult to separate their personality from hypomania and that they may still take risks during a euthymic mood state but they would be more calculated and less impulsive.

Risk-taking behaviours related to being ‘manic’, ‘high’, ‘unwell’ or ‘hypomanic’ were described by the majority of PWLE participants (*n = *17/18, 94%) as were risk-taking behaviours associated with depression (*n = *13/18, 72%). Some participants also described risk-taking behaviours during mixed (*n = *5/18, 285) and rapid cycling (*n = *2/18, 11%) mood states. The types of risk-taking which participants associated with an elevated mood included the use of dating apps to meet with people for sexual encounters during a manic or hypomanic episode, having affairs, spending large amounts of money on unusual items or giving away money and belongings to strangers, participating in extreme sports like paragliding and open water swimming, and going out at night alone to unusual places (see Table 2 and Supplementary Material S1 (2.1, Table S1) for the full classification of behaviours described by participants). When talking about periods of depression, participants described risk-taking behaviours associated with self-harm, suicidal ideation, overdosing or suicide attempts.

The results from the USAS semantic tagger presented in Table 5 demonstrate that the PWLE group were more likely to use semantic terms within the domain of anatomy and physiology, religion, the past tense, negative words and vehicles. Meanwhile, the PWLE group were less likely to use semantic terms associated with danger, cause and effect, and success and failure.

**Table 5.** Overused and underused semantic domains in the PWLE group corpus (using the HP corpus as a reference).

**ITEM**	**O1**	**%1**	**O2**	**%2**		**LL**	**%DIFF**	**USAS tag descriptor**
**Overused domains (PWLE group compared to HP group)**								
**Z8**	16329	18.51	2568	14.61	+	131.25	26.66	Pronouns
**T1.3**	509	0.58	33	0.19	+	54.73	207.25	Time: period
**B1**	371	0.42	18	0.1	+	53.62	310.57	Anatomy and physiology
**S9**	130	0.15	0	0	+	47.23	1.47346E+17	Religion and the supernatural
**N1**	662	0.75	59	0.34	+	43.93	123.51	Numbers
**A14**	814	0.92	93	0.53	+	29.88	74.35	Exclusivisers/particularisers
**M1**	993	1.13	121	0.69	+	29.61	63.48	Moving, coming and going
**T1.1.1**	313	0.35	23	0.13	+	28.55	171.08	Time: past
**S2.2**	90	0.1	1	0.01	+	25.28	1692.8	People: male
**Z6**	1896	2.15	279	1.59	+	24.03	35.37	Negative
**E2+**	322	0.36	27	0.15	+	23.87	137.56	Like
**M3**	205	0.23	15	0.09	+	18.81	172.24	Vehicles and transport on land
**T1**	303	0.34	28	0.16	+	18.73	115.56	Time
**X2.2+**	680	0.77	85	0.48	+	18.5	59.36	Knowledgeable
**K2**	42	0.05	0	0	+	15.26	4.76039E+16	Music and related activities
**Underused domains (PWLE group compared to HP group)**								
**A15-**	408	0.46	224	1.27	–	130.65	−63.72	Danger
**S2**	544	0.62	263	1.5	–	123.05	−58.8	People
**S5+**	98	0.11	92	0.52	–	102.7	−78.78	Belonging to a group
**S1.1.1**	178	0.2	104	0.59	–	66.76	−65.91	Social actions, states and processes
**A2.2**	298	0.34	131	0.75	–	50.62	−54.69	Cause and effect/connection
**G1.1**	39	0.04	40	0.23	–	48.27	−80.58	Government
**N5++**	288	0.33	126	0.72	–	48.19	−54.47	Quantities: many/much
**S8+**	329	0.37	137	0.78	–	46.89	−52.16	Helping
**X3.4**	227	0.26	104	0.59	–	43.81	−56.52	Sensory: sight
**A1.5.1**	59	0.07	45	0.26	–	40.71	−73.88	Using
**A1.3-**	15	0.02	21	0.12	–	31.94	−85.77	No caution
**N6+**	199	0.23	85	0.48	–	30.83	−53.36	Frequent
**X9.2**	19	0.02	21	0.12	–	26.95	−81.98	Success and failure
**N6-**	38	0.04	29	0.17	–	26.25	−73.9	Infrequent
**A4.2+**	95	0.11	48	0.27	–	24.34	−60.58	Detailed

PWLE: people with lived experience; HP: healthcare professional; USAS: UCREL semantic analysis system.

O1 is observed frequency in PWLE group corpus.

O2 is observed frequency in HP group corpus.

%1 and %2 values show relative frequencies in the texts.

+ indicates overuse in O1 relative to O2.

– indicates underuse in O1 relative to O2.

The table is sorted on log-likelihood (LL) value.

The LL cut-off value was set to 3.83, thus the results are significant at the level of *p* < 0.05.^67^

%DIFF indicates the proportion (%) of the difference between the normalised frequencies of a word in two corpora (or sub-corpora).^68^

##### Keywords from top 10 risk-taking behaviours

Table 6 shows the top 10 keywords for the most-cited risk-taking behaviours by the PWLE group (based on the behaviours which were cited by the highest number of participants) which demonstrates the types of topic that were talked about within each behaviour.

**Table 6.** Keywords from the top 10 most frequent risk-taking behaviours for PWLE using a sub-corpus of only the risk-taking behaviour text.^67,68^

Behaviour	Item	Frequency (focus)	Frequency (reference)	Relative frequency (focus)	Relative frequency (reference)	DOCF (focus)	DOCF (reference)	ALDF (focus)	Score
Spending money excessively									
	*impulsive*	3	2	3205.13	0.17	1	2	1.45	2742.58
	*overspend*	3	3	3205.13	0.25	2	3	1.60	2557.68
	*spending*	5	94	5341.88	7.94	4	81	3.65	597.38
	*debt*	6	157	6410.26	13.27	6	79	3.98	449.34
	*risk*	3	380	3205.13	32.11	3	186	1.97	96.82
	*shopping*	3	530	3205.13	44.79	3	282	2.66	70.02
	*bill*	3	566	3205.13	47.83	1	225	1.15	65.66
	*spend*	9	2567	9615.38	216.94	7	771	7.54	44.13
	*expensive*	3	1558	3205.13	131.67	2	593	2.13	24.17
	*buy*	13	7462	13888.89	630.61	5	1018	8.13	21.99
Going to dangerous or unusual places									
	*bipolar*	3	13	4418.26	1.10	1	6	1.09	2105.79
	*guard*	3	150	4418.26	12.68	1	83	1.17	323.13
	*normally*	3	1426	4418.26	120.51	1	616	1.42	36.37
	*night*	3	5535	4418.26	467.76	2	954	2.24	9.43
	*or*	14	41,630	20618.56	3518.15	5	1251	7.57	5.86
	*might*	3	10,314	4418.26	871.64	2	1185	2.25	5.06
	*where*	5	17,602	7363.77	1487.54	3	1228	3.12	4.95
	*very*	5	18,339	7363.77	1549.83	3	1213	3.34	4.75
	*something*	5	19,344	7363.77	1634.76	3	1235	4.26	4.50
	*kind*	3	12,164	4418.26	1027.98	2	1119	1.93	4.30
Suicidal ideation									
	*suicidal*	9	13	15332.20	1.10	7	13	6.58	7306.29
	*suicide*	9	104	15332.20	8.79	5	50	5.52	1566.37
	*thought*	6	575	10221.46	48.59	4	368	4.48	206.13
	*pain*	3	575	5110.73	48.59	2	282	1.95	103.07
	*feel*	3	7748	5110.73	654.78	2	1064	2.07	7.80
	*want*	5	24,318	8517.89	2055.11	2	1238	2.39	4.14
	*because*	4	24,893	6814.31	2103.70	3	1226	2.96	3.24
	*as*	5	31,473	8517.89	2659.78	2	1247	1.99	3.20
	*about*	5	33,079	8517.89	2795.50	4	1247	3.17	3.05
	*thing*	4	30,344	6814.31	2564.37	2	1246	2.71	2.66
Having ‘no filter’ or a lack of social boundaries									
	*information*	3	613	3856.04	51.80	1	317	1.24	73.04
	*past*	3	2162	3856.04	182.71	2	735	2.79	21.00
	*too*	7	8135	8997.43	687.49	3	1158	4.53	13.07
	*people*	13	23,032	16709.51	1946.43	5	1221	8.22	8.58
	*anyway*	3	6596	3856.04	557.43	2	1107	2.09	6.91
	*tell*	5	11,117	6426.74	939.50	3	1186	3.10	6.83
	*very*	7	18,339	8997.43	1549.83	5	1213	3.41	5.80
	*talk*	3	8273	3856.04	699.15	2	1124	1.66	5.51
	*other*	4	13,223	5141.39	1117.47	1	1213	1.72	4.60
	*thing*	8	30,344	10282.78	2564.37	3	1246	6.22	4.01
									
Excessive generosity									
	*maxed*	3	3	2570.69	0.25	1	3	1.12	2051.56
	*quid*	16	1805	13710.37	152.54	2	483	4.15	89.30
	*credit*	3	401	2570.69	33.89	2	158	2.37	73.71
	*church*	5	1016	4284.49	85.86	1	264	1.65	49.34
	*bank*	3	1018	2570.69	86.03	2	342	2.03	29.55
	*woman*	6	2605	5141.39	220.15	2	692	2.75	23.25
	*give*	16	9735	13710.37	822.70	3	1163	7.77	16.65
	*birthday*	3	1824	2570.69	154.15	2	501	1.29	16.58
	*spend*	4	2567	3427.59	216.94	3	771	2.24	15.73
	*money*	10	6667	8568.98	563.43	4	940	5.31	15.18
Self-harm									
	*self-harm*	7	1	13133.21	0.08	4	1	3.54	12110.73
	*self-harmed*	3	0	5628.52	0.00	3	0	2.30	5629.52
	*self-harming*	3	6	5628.52	0.51	3	3	2.98	3735.43
	*impair*	3	16	5628.52	1.35	1	12	1.14	2393.34
	*risk*	3	380	5628.52	32.11	2	186	2.03	170.01
	*myself*	9	1773	16885.55	149.84	5	692	5.60	111.95
	*cut*	5	2138	9380.86	180.68	4	652	3.36	51.64
	*by*	3	8773	5628.52	741.41	1	1163	1.14	7.58
	*because*	6	24,893	11257.04	2103.70	4	1226	5.37	5.35
	*sort*	3	16,142	5628.52	1364.16	3	1152	2.60	4.12
Hypersexuality (not specified)									
	*sexuality*	3	10	6451.61	0.85	2	9	2.36	3497.16
	*risky*	3	57	6451.61	4.82	2	37	1.95	1109.26
	*sex*	6	506	12903.23	42.76	3	175	3.32	294.87
	*definitely*	3	3258	6451.61	275.33	1	830	2.17	23.35
	*many*	4	5060	8602.15	427.62	2	1058	1.85	20.07
	*feel*	3	7748	6451.61	654.78	2	1064	1.79	9.84
	*before*	3	7771	6451.61	656.73	2	1153	1.98	9.81
	*more*	3	16,177	6451.61	1367.12	2	1221	2.10	4.72
	*could*	4	22,284	8602.15	1883.22	1	1238	1.16	4.57
	*put*	3	17,118	6451.61	1446.64	2	1206	2.67	4.46
Aggression, violence or destruction									
	*hit*	6	1217	10526.32	102.85	4	472	3.31	101.37
	*throw*	5	1254	8771.93	105.98	3	521	3.15	82.01
	*glass*	4	1312	7017.54	110.88	3	451	2.84	62.73
	*wall*	3	1033	5263.16	87.30	3	412	2.49	59.62
	*anyone*	3	1793	5263.16	151.53	1	699	1.50	34.51
	*phone*	3	4178	5263.16	353.08	1	779	1.37	14.87
	*first*	3	7813	5263.16	660.28	3	1145	2.84	7.96
	*down*	3	13,143	5263.16	1110.71	2	1189	1.81	4.74
	*time*	4	21,533	7017.54	1819.75	4	1240	2.43	3.86
	*people*	4	23,032	7017.54	1946.43	3	1221	2.79	3.60
Dangerous driving									
	*motorbike*	5	108	6002.40	9.13	2	62	3.20	592.81
	*potential*	3	120	3601.44	10.14	1	93	1.20	323.34
	*driving*	4	212	4801.92	17.92	2	132	2.02	253.91
	*fast*	8	762	9603.84	64.40	5	409	4.51	146.87
	*lane*	3	295	3601.44	24.93	1	129	1.22	138.93
	*drive*	12	3601	14405.76	304.32	6	739	5.92	47.19
	*road*	8	2874	9603.84	242.88	3	656	5.37	39.38
	*ring*	3	1859	3601.44	157.10	1	551	1.64	22.79
	*high*	3	2020	3601.44	170.71	1	692	1.66	20.98
	*car*	3	5164	3601.44	436.41	2	805	2.03	8.24
Excessive alcohol									
	*alcohol*	8	391	22662.89	33.04	4	173	3.48	665.74
	*depress*	3	195	8498.58	16.48	2	125	1.95	486.26
	*beer*	3	944	8498.58	79.78	1	275	1.95	105.22
	*drink*	9	3570	25495.75	301.70	5	712	8.33	84.23
	*night*	4	5535	11331.44	467.76	2	954	2.87	24.18
	*too*	3	8135	8498.58	687.49	1	1158	2.08	12.35
	*much*	3	12,949	8498.58	1094.32	1	1208	2.08	7.76
	*down*	3	13,143	8498.58	1110.71	2	1189	2.67	7.65
	*use*	3	15,857	8498.58	1340.07	2	1193	2.28	6.34
	*very*	3	18,339	8498.58	1549.83	2	1213	2.44	5.48

DOCF: document frequency; ALDF: average logarithmic distance frequency.

These keywords show how risk-taking behaviours were talked about by people living with bipolar, for example, the word ‘suicidal’ was used by *n = *7 of the participants within the behaviour of suicidal ideation, and ‘cut’ was used by *n = *4 participants when talking about self-harm. These keywords also provide some insight into the impact that risk-taking behaviours can have, for example, the word ‘debt’ was referenced by *n = *6 participants within the behaviour of spending money excessively.

##### Positive risk-taking

The majority (*n = *15/18, 83%) of PWLE interview participants defined risk-taking as doing something which would ‘interrupt personal safety’ or put yourself ‘in a position of danger’. However, participants were also asked whether there were any positive outcomes associated with risk-taking, to which *n = *9/15 participants agreed (60%). Participants described having increased confidence during hypomania which led to positive risk-taking like applying for jobs that they otherwise wouldn’t have done, meeting new people and taking part in new activities.

Within the HP group, all participants identified specific types of risk-taking behaviour which could have a damaging impact, but some participants also described that risk-taking is something that can be encouraged in situations where it may have a positive outcome (in relation to positive risk-taking from a clinical perspective, e.g.^70^).

#### The impact of risk-taking behaviours

PWLE participants described a number of ways in which risk-taking had impacted their lives, including the impact of suicide attempts on relationships, debt, traffic accidents, unplanned pregnancy, anxiety around sexually transmitted diseases, the effect of verbal aggression on family members, the effect of violence towards family members, loneliness and being in vulnerable situations where the potential for harm occurring is increased. HPs reported impacts such as bankruptcy, homelessness and the breakdown of relationships. Table 7 provides qualitative evidence from concordance lines for the type of impacts described during interviews.

**Table 7.** Interview excerpts demonstrating the impacts of risk-taking behaviours.

Participant	Quote	Group
P09	‘*The suicide attempts have had a massive impact on my family and trust, they were understandably devastated.*’	PWLE
P11	‘*I’m in debt now because of the spending, but I also worried about sexually transmitted infections and unwanted pregnancy. If I’m hypersexual I’m more likely to take risks with protection.*’	PWLE
P13	‘*I think just being vulnerable, being in certain situations, things like with men you don’t know and you don’t know what's going to happen and you might get attacked or assaulted.*’	PWLE
P15	‘*The biggest impact is that I hit my wife during an episode.*’	PWLE
P23	‘*I’ve worked with people who’ve had to go bankrupt. Other people become homeless or get very close to it, relationships end and marriages break down. I think these are really some of the most severe outcomes from any mental health problem.*’	HP

PWLE: people with lived experience; HP: healthcare professional.

#### Emotions related to risk-taking

PWLE participants were asked to describe their emotions related to risk-taking – both while undertaking something risky and then in hindsight. Table 8 shows that *n = *6/18 (33%) PWLE participants used an analogy of having a superpower, being ‘invincible’ or ‘immortal’ to describe how they felt when doing something risky. They described feeling as though no harm could come to them and that ‘you can take anything on’. Participants described that they were less likely to think about the negative consequences of risk-taking behaviour in the moment and *n = *13/18 (72%) described that they were ‘caught up’ in feeling confident, excited, euphoric or elated. When reflecting on risk-taking behaviour after it had occurred, *n = *6/18 (33%) of participants described feelings of shame, regret and relief that no harm came to them.

**Table 8.** KWIC concordance lines from PWLE participant corpus for the tokens of ‘superpower’, ‘superman’, ‘invincible’ and ‘immortal’ _(80 character limit for the character width but normalised to include full words)._

**REFERENCE**	**LEFT**	**KWIC**	**RIGHT**
**P03**	having your own	**Superpower**	and loads of energy
**P08**	I’ve heard it referred to as	**Superman**	Syndrome, basically, so I did think I
**P15**	You know, you feel like	**Superman**	.’
**P03**	because you are totally	**Invincible**	in that moment. And utterly convicted
**P07**	I was just saying, it's either	**Invincible**	or nihilistic. Yeah, just total
**P08**	basically, so I did think I was	**Invincible**	.’ So I was on dating apps, they
**P11**	danger whatsoever. I think I’m	**Invincible**	.’ So if I was to risk take it would
**P05**	Well, I suppose you just feel	**Immortal**	and when you’re high you don’t

KWIC: key word in context; PWLE: people with lived experience.

Using the USAS tagger, we also observed that when talking about risk-taking behaviours the PWLE group overused terms within the domains of fear/shock, sad and worry demonstrating that these types of emotions could potentially be under-explored or under-recognised by HPs.^e^ These domains are shown in Table 9.

**Table 9.** Significant USAS overused emotion tags in PWLE compared to HP group corpus.

**Item**	**O1**	**%1**	**O2**	**%2**		**LL**	**%DIFF**	**USAS tag descriptor**
**E2+**	322	0.36	27	0.15	+	23.87	137.56	Like
**E5–**	83	0.09	4	0.02	+	12.07	313.34	Fear/shock
**E4.1–**	177	0.2	18	0.1	+	8.87	95.88	Sad
**E6–**	114	0.13	12	0.07	+	5.25	89.24	Worry

PWLE: people with lived experience; HP: healthcare professional; USAS: UCREL semantic analysis system.

#### Access to support

##### Routine support for risk-taking behaviours

Participants were asked a number of questions relating to the support they receive relating to risk-taking behaviours from HPs. When asked whether HPs routinely ask about risk-taking behaviours, *n = *9/18 (50%) of the PWLE participants described that risk-taking behaviours were not routinely asked about during their healthcare appointments, or only asked about in the context of specific behaviours such as self-harm, suicide and spending. Two (10%) participants stated that HPs did ask about risk-taking behaviours but they didn’t like to talk about their experiences for fear of being judged, based on their previous negative experiences. Two (10%) participants stated that they were routinely asked about their risk-taking behaviours (*n = *1 of these participants was referring to psychiatry that they paid for privately). Table 10 demonstrates participant's experiences with being asked about risk-taking behaviours.

**Table 10.** Interview excerpts demonstrating participants’ experience of routine support for risk-taking behaviours.

Participant	Quote	Group
P12	‘*They didn’t ask anything about risk-taking from what I remember. Though, I did talk about one incident and they just kind of dismissed it.*’	PWLE
P14	*‘They seem to ask, are you going to kill yourself or anyone else, which is obviously an important question to ask. But it shouldn’t be the only question. Sometimes I could have a bad argument with someone and I could cause them real emotional damage. Risk-taking is a lot more subtle than just self-harm and suicide.*’	PWLE
P05	‘*When I go and see a psychiatrist, all they seem to be interested in is working out how much medication I should take to control my illness.*’	PWLE
P02	‘*I’m lucky to have found a great psychiatrist through lots of trial and error, and they will ask me about anything and everything, but I do pay for that privately.*’	PWLE
P09	‘*In a longer appointment they will ask about a lot more types of risk-taking behaviour, if it's just a triage then it's mainly about self-harm and thoughts of suicide.*’	PWLE

PWLE: people with lived experience.

##### What support do participants want?

Participants (*n = *15/18, 83%) overwhelmingly described that they would like more access to support for risk-taking behaviours. Some participants suggested specific methods of support, including more comprehensive care plans, advice about how to take risks safely (e.g. in the context of extreme sports), more diversity in psychiatric teams, dedicated mental health professionals in GP surgeries, a safe place to go to with mental health nurses and without enforced medication as an alternative to hospitalisation, and less judgment from HPs. Three (17%) of these participants also stated that they were unsure what extra support would look like in the context of an elevated mood ‘when you are convinced that what you’re doing is right’ and that they would be unlikely to proactively seek out support in a manic or hypomanic episode. Ten PWLE participants (55%) reported that they would like improved access to psychological therapies, *n = *3(17%) participants described only speaking to a psychiatrist to review their medication or during very brief telephone appointments every few months, *n = *2 (11%) participants reported that they had relapsed due to long wait-times for an appointment with their healthcare provider, and *n = *2 (11%) participants reported that services had become more fragmented and difficult to access since the Covid-19 pandemic. Three HP participants (60%) also described that there is not currently enough support provided by healthcare services for risk-taking behaviours. Two out of 18 (11%) of PWLE participants described their experiences of stigma when seeking support for their risk-taking behaviours.

Table 11 provides qualitative evidence for previous experiences with accessing support or the type of support that participants feel should be available.

**Table 11.** Interview excerpts demonstrating the further support that participants would like.

Participant	Quote	Group
P12	‘*I’ve tried community support programmes but I didn’t find them helpful. I went to a group session and talked about something I’d done and someone called me stupid. I want sensible advice, I don’t want to go somewhere and receive abuse. It's very personal to talk about some of the risks you’ve taken, you don’t want to do that in front of a room full of people*.’	PWLE
P10	‘*I think there should be a GP in every practice with specialist mental health knowledge, that would be ideal.*’	PWLE
P13	‘*With risk taking I’d prefer to talk to a female. It's very intimate talking about sexual experiences and sometimes you can feel looked down on as a woman.*’	PWLE
P11	‘*You’re supposed to have a care plan, but there's a big difference between what you have and what you’re supposed to have. I would like strategies in place that I could use when I’m on the way to becoming unwell, or I would like to be able to involve a Community Psychiatric Nurse who I could talk to. At the moment I have a psychiatrist who I only see every three months or something silly*.’	PWLE
P13	‘*I would like healthcare professionals to understand that I don’t just want to take drugs, sleep around and rip all my clothes off. I want them to understand that this is an escalating process, and medicating me out of that and treating those symptoms like they are the main issue doesn’t help, it's just an outlet for how I’m feeling at the time.*’	PWLE
P15	‘*Well the support that I would have liked over the years is to get more black doctors who understood*.’	PWLE
P02	‘*To be honest I have no idea what that support would look like. At the point when you need critical help you’ve probably already done something risky. What are they going to do, tie me down?*’	PWLE
P12	‘*I think there's a huge lack of awareness about what people get up to. There should be some acknowledgement of these behaviours but I don’t necessarily want to be medicated out of them. It would be good to explore ways of doing things which would satisfy that kind of wanderlust and quest to excitement but with less danger.*’	PWLE
P23	‘*There's absolutely not enough support, it's appalling, some of these things can destroy lives. The access to therapy generally for bipolar is patchy, and not enough people get offered therapy.*’	HP

PWLE: people with lived experience; HP: healthcare professional.

##### Utility of a risk-taking measurement tool

PWLE participants all responded positively to the risk-taking questionnaire and there was no negative feedback on the use of such a tool. PWLE participants described that the tool helped them reflect on their risk-taking behaviours objectively and that a structured list made it easier to remember specific events or behaviours that they might not have otherwise remembered, with many participants stating that they would be happy to use a similar tool during consultations and then talk through their responses to the questionnaire with their HP.

The majority of the HP group also viewed the tool positively (*n = *4/5, 80%), describing that it would be useful as a prompt for discussion and also when formulating a treatment plan, and *n = *4/5 (80%) of the HP group also stated that they had not seen a risk-taking measurement tool like this before in the context of clinical practice. One HP participant (20%) reported that this type of questionnaire could be triggering or insulting to people with a diagnosis of bipolar, suggesting that it would be better to allow the individual to talk about these behaviours voluntarily. Table 12 provides qualitative evidence demonstrating participants’ responses to using a risk-taking measurement tool.

**Table 12.** Interview excerpts demonstrating participants’ attitudes towards the risk-taking questionnaire.

Participant	Quote	Group
P01	‘*I think it would be good to complete the questionnaire and then talk about it. I think when I was assessed the psychiatrist probably did cover a lot of those behaviours but I didn’t have time to sit down with a questionnaire and think about my answers.*’	PWLE
P12	‘*I’d be very happy to complete a questionnaire like that in the future, it felt non-judgmental and made it easier to talk about my experiences.*’	PWLE
P13	‘*It would make me think about things more. Rather than a doctor saying this is your diagnosis and these are your symptoms, here is some safeguarding to prevent these risks – this would make it into more of a discussion. I think we need therapeutic based conversation, especially when you’re vulnerable. You don’t want to feel guilty or ashamed by talking about things.*’	PWLE
P17	‘*This is good because it gets to the core of some behaviours. It made it easier to articulate my memories and triggered me to remember some things that otherwise would have been very difficult to talk about.*’	PWLE
P23	‘*It's easier to provide a list which people can look at and identify possible risk-taking behaviours rather than expecting service users to volunteer information, which they might not do because of shame. Creating a list like this is normalising and de-shaming if it is delivered in the right way.*’	HP

PWLE: people with lived experience; HP: healthcare professional.

### Summary of results

The results presented here demonstrate that PWLE consider risk-taking behaviours to be a fundamental aspect of changing mood states, and for the HP group risk-taking seems to align particularly closely with suicidal behaviours. The analysis demonstrates that the impact of risk-taking behaviours can be severe and long-lasting, and that both groups of participants describe the need for greater provision of psychologically informed care. All of the PWLE group and the majority of the HP group recognised the utility of a standardised questionnaire as an elicitation tool to talk about risk-taking, reporting that it would be useful in conjunction with a ‘therapeutic-based’ conversation about risk-taking.

## Discussion

This study presents the first step towards an understanding of risk-taking behaviours where lived experience is foregrounded, building upon the previous work in this area which has predominantly relied on behavioural and laboratory tasks and self-report measures which have not been validated specifically for bipolar. This work is clinically relevant because it has identified that some risk-taking behaviours which are ‘taboo’, such as sexual risk-taking, are not talked about enough during consultations with HPs, a finding which has also been reported elsewhere in the literature.^71,72^ We have identified a number of ways in which clinical support could be improved in relation to risk-taking behaviours, including increased access to psychologically informed care for all people diagnosed with bipolar, increased diversity within the healthcare workforce, the development of formulated care plans specific to the behaviours presented by individuals and the development of a risk-taking elicitation tool which is specific to bipolar.

Our classification system (section ‘Suggested classification system for risk-taking behaviours’) presents a unified list of risk-taking behaviours based on empirical evidence which includes, extends and updates those behaviours currently documented in available risk-taking behaviour measures (a synthesised list is provided in Supplementary Material S1 (2.3, Tables S2 and S2.4, Table S3)).^f^ The evidence from both the risk-taking questionnaire and the narrative interviews (sections ‘The risk-taking questionnaire (modified MIS-CAM)’ and ‘Corpus analysis and qualitative evidence for key interview themes’) suggests that HPs generally demonstrate knowledge of the types of risk-taking that people living with bipolar engage in, although specific behaviours such as taking recreational drugs and attempting suicide may be overrepresented and other behaviours such as breaking social boundaries, excessive eating and extreme sports may be underrepresented. Participant responses suggest that HPs may only routinely assess the presence of a limited number of risk-taking behaviours, resulting in opportunities for intervention being missed. The reasons for this may include prioritising the most severe forms of risk-taking due to time and/or financial constraints in order to have the most direct impact on saving lives (in the case of self-harm and suicide), assuming that a service user will be forthcoming in talking about a risk-taking event when in fact the individual may feel uncomfortable doing so,^71^ having a genuine lack of awareness around the types of risk-taking behaviour that people engage in aside from those most commonly cited in the literature, or feeling uncomfortable broaching taboo topics such as sex and eating habits.^73^ The impacts from risk-taking behaviours as demonstrated by the narratives within this study can be life-altering, including pregnancy, bankruptcy and potentially life-threatening injuries resulting from dangerous driving or risky behaviours like standing on bridges. There are also more subtle risks like ‘having no filter’ around friends or colleagues, initiating relationships with strangers or giving away household belongings, which may be the type of behaviours which are not routinely discussed but can still have a serious impact on a person's life.

Analysis of the data related to access to support (section ‘Access to support’) shows that the majority of PWLE participants wanted more access to support for risk-taking behaviours, or they would have liked more support for these behaviours at earlier stages of their diagnosis. PWLE participants demonstrated good insight into their risk-taking behaviours and many of the participants clearly distinguished between behaviours that they engaged in during episodes of hypomania and mania or depression (although some respondents reported rapid-cycling or dysphoric mania where risk-taking behaviours are less easily defined between mood states). This distinction between mood states and the associated risk-taking behaviours should therefore be integrated routinely within a care plan so that service users can work with HPs to proactively develop management strategies for specific risk-taking behaviours that could be implemented at the earliest signs of an elevated or depressed mood state, for example, blocking dating app use on a smartphone or restricting access to social media accounts to avoid posting something that the individual may later regret. The psychosocial approach of formulation is particularly relevant here, i.e. implementing therapeutic solutions which are formulated based on an individual's symptoms, problems and circumstances, rather than a diagnostic code.^74^ As described by Lukacs et al.,^23^ risk-taking is currently auxiliary to treatments in bipolar and the targeted mechanisms of risk-taking are often biological rather than behavioural, although a behavioural approach could be of benefit. PWLE participant experiences with the healthcare system varied widely and suggest that a standardised pathway for treatment of bipolar in general and for risk-taking behaviours as part of this could improve the detection, intervention and reporting for risk-taking, thus providing more empirical data which could help to normalise people's experiences and remove some of the stigma and shame that is felt as a repercussion of risk-taking behaviours.^1^ Despite the recommendations of the NICE guidelines^75^ which state that all people with a diagnosis of bipolar should be offered psychological intervention, audits conducted by South London and Maudsley NHS Foundation Trust and Manchester Mental Health and Social Care Trust show that rates of access to psychological interventions for eligible individuals with severe mental range between only 7 and 10%.

As demonstrated in section ‘What support do participants want?’, some PWLE participants reported feeling judged by their HPs for describing risky behaviours, or that they refrained from telling their healthcare team about risk-taking for fear of being ‘medicated’ out of these behaviours. Responses also highlighted issues related to workforce diversity. One female participant described that the majority of psychiatrists they had been treated by were ‘older, white men’, and that they would feel more comfortable discussing their experiences of hypersexuality with a female HP. Although the RCPsych (Royal College of Psychiatrists) report that the proportion of female consultants is increasing (48.2% female and 51.8% male psychiatrists in 2021), the proportion of male-to-female psychiatrists varies considerably between the devolved nations and also regionally within England.^76^ Similarly, another PWLE participant described that the majority of HPs they have interacted with are white, and that they would prefer to talk to someone who shares their own ethnicity as a shared cultural understanding may improve the support they receive. The BPS (British Psychological Society) states that ‘it is long acknowledged that there is a lack of psychiatrists and psychologists of ethnic backgrounds within mental health professions’ and that ‘an ethnically diverse and representative workforce within psychiatry and psychology professions is imperative for addressing the well-documented unequal clinical outcomes and overall negative experiences that BAME mental health service users face’.^77^ The results presented in section ‘What support do participants want?’ also demonstrate that some participants were unsure what type of support could be provided for risk-taking behaviours as they didn’t believe that they would access support when they were in an elevated mood due to the positive feelings from being ‘high’ and the ‘superman syndrome’ that can accompany this (see results from section ‘Emotions related to risk-taking’). There is some evidence from cognitive behavioural therapy studies which suggests that formulating care plans in the service user's own words during euthymic periods can be effective in helping the individual to practice management strategies after a mood change.^78^

The development of risk management strategies relies on risk-taking behaviours being systematically identified prior to a service user becoming unwell, and the evidence from these interviews seems to suggest that this is only routinely happening for a restricted number of behaviours. If HPs were to routinely conduct a checklist-type exercise similar to the modified MIS-CAM tool, participants may find it easier to identify and report their risk-taking behaviours that they may otherwise feel uncomfortable discussing, either due to associated stigma or because they are unsure whether it qualifies as a ‘valid’ risk-taking behaviour. The results from section ‘Utility of a risk-taking measurement tool’ demonstrate that participants responded positively to the risk-taking questionnaire and many participants stated that they would find it helpful to complete such a questionnaire with their healthcare team and then have the opportunity to talk through any behaviours identified. Although *n = *1 (20%) participant in the HP group suggested that such a tool could be triggering for service users, this sentiment was not echoed by the PWLE participants. We believe that the evidence provided in this study demonstrates that a risk-taking questionnaire which is designed specifically in relation to bipolar would be of benefit to people living with the diagnosis and could be used as an elicitation tool during consultations.

In future research, we intend to scale-up the results from our interview study using data from Reddit users who self-report a diagnosis of bipolar and who also talk about risk-taking behaviours. By assessing how people talk about risk-taking in an unstructured setting such as social media, we hope to provide quantitative evidence on a large scale to demonstrate the prevalence of specific risk-taking behaviours, using data from a typically hard-to-reach population on a topic which may otherwise be underrepresented in clinical practice. Research suggests that the anonymity afforded by social media sites such as Reddit may make people more likely to self-disclose ‘things they are unwilling to do or say in face-to-face settings’ and enable online users to talk freely about content such as mental health struggles, self-harm behaviours and sexual taboos.^79^ Previous studies have successfully utilised Natural language processing (NLP) methods on social media and electronic health record (EHR) data specifically in the study of bipolar for various tasks, for example, detecting a diagnosis from Reddit data,^80^ predicting how emotional states change after Reddit interactions,^81^ and using the text within medical records to measure health outcomes of people with a diagnosis of bipolar.^82,83^

It is also important to address the limitations of this study so that future work can build and improve upon these areas. In terms of recruitment, the following aspects are likely to have resulted in some level of bias: we recruited only for PWLE participants in a euthymic mood state, the majority of the PWLE participants recruited were female (14/18, 77%), and the majority of the HP participants were male (4/5, 80%). Regarding study design, interviews are prone to bias from demand characteristics^84^ and the risk-taking behaviours were annotated primarily by one researcher (DH), with second annotations being provided for 40% of the interviews by a second researcher (PR). Also, there is currently no validated risk-taking measure available specifically for bipolar. Although we conducted test interviews, we are unable to report on the validity of the risk-taking questionnaire that we used and recognise that this is an important avenue for further research. Finally, in order for the field to continue to learn more about the prevalence of risk-taking behaviours, there needs to be collaboration between the psychosocial and biomedical domains so that we can better understand not only what causes people to participate in risky behaviour but also to formulate individualised care plans which provide pragmatic management strategies. Using large-scale data such as social media together with innovative methods from NLP, we can build an empirical evidence base to learn from, and in turn develop theories and best practice which are based on lived-experience data.

## Conclusion

This study has used a mixed-method multi-method design to foreground lived experience accounts of risk-taking in bipolar, presenting a classification system of 39 risk-taking behaviours. We have provided qualitative and quantitative evidence to demonstrate that people living with bipolar engage in a wide range of risk-taking behaviours across different mood states which can severely affect the lives of these individuals and those people around them. This research has also demonstrated the practical application of computational linguistic methods to health research to provide a more efficient process for analysing textual data and identifying key insights. The evidence highlights that more support is wanted and needed to help manage risk-taking behaviours and that a standardised approach to assessing and monitoring behaviours, such as a risk-taking questionnaire, may be beneficial. Much of the literature on the topic of risk-taking in bipolar has thus far relied on behavioural and laboratory exercises or surveys and measures which are not validated specifically for bipolar. There needs to be a greater emphasis in future work on incorporating lived experience data into these measures which assess risk-taking, as well as a drive towards understanding *what* behaviours people report instead of focusing predominantly on *why* they engage with these behaviours. Future work should aim to investigate risk-taking behaviours at scale using lived experience accounts to determine the extent to which some of these behaviours are under-explored in clinical practice and to devise pragmatic intervention strategies which are formulated on a case-by-case basis.

## Supplemental Material

sj-docx-1-dhj-10.1177_20552076241269580 - Supplemental material for Lived experience at the core: A classification system for risk-taking behaviours in bipolar Supplemental material, sj-docx-1-dhj-10.1177_20552076241269580 for Lived experience at the core: A classification system for risk-taking behaviours in bipolar by Daisy Harvey, Paul Rayson, Fiona Lobban, Jasper Palmier-Claus and Steven Jones in DIGITAL HEALTH

sj-docx-2-dhj-10.1177_20552076241269580 - Supplemental material for Lived experience at the core: A classification system for risk-taking behaviours in bipolar Supplemental material, sj-docx-2-dhj-10.1177_20552076241269580 for Lived experience at the core: A classification system for risk-taking behaviours in bipolar by Daisy Harvey, Paul Rayson, Fiona Lobban, Jasper Palmier-Claus and Steven Jones in DIGITAL HEALTH

## Notes

a. Corpus linguistics is a branch of computational linguistics which relies on “real-world instances of language use in order to…explore trends about the ways in which people actually produce language”, and can be used to explore phenomena and quantify patterns.^85^ The majority of corpus methods are based on the use of statistics to allow the comparison of frequencies and can be used to analyse not only the words within the corpus (dataset of text or language), but also the annotations that may be applied to these words, such as semantic domains or Part-of-Speech tags.

b. The MDQ^48^ yielded good sensitivity (0.73) and very good specificity (0.90) in,^48^ the Altman mania scale has demonstrated good validity in the inpatient setting to screen for the presence of and/or stage the severity of manic symptoms,^49^ and the Self-report Quick Inventory of Depressive Symptomatology (QIDS-SR16) has been reported as a reliable and valid self-report scale in recent research.^86^

c. Professor Evans has kindly provided permission to adapt the MIS and CAM tools for use in this research.^16^ Future researchers who wish to make similar adaptations should contact Professor Evans for authorization, see: https://www.psyctc.org/psyctc/root/tools/mis/

d. Regarding the psychometric properties of the MIS and the CAM, the authors^16^ state in the original release of the tools that “this paper does not present detailed psychometric data on these measures as we believe they are clinically useful irrespective of those nomothetic considerations.”

e. The domain of ‘like’ was also significantly overused by the PWLE group but this was mainly attributed to the frequent use of the word ‘like’ as a filler word.

f. Some behaviours taken from the DOSPERT^9,10^ which appear in the synthesised list available in SM_1 2.4, Table 3 have not been included, such as ‘sunbathing without sunscreen’ (because we think this is something that a lot of people will do regardless of whether or not they have bipolar), and ‘not returning a wallet that you found’, ‘taking some questionable deductions on your income tax return’, ‘passing off somebody else's work as your own’, ‘revealing a friend's secret to someone else’, and ‘admitting your tastes are different from a friend’ because these are all hypothetical moral or ethical risks which were not cited by any of our participants and represent a different dimension of risk-taking behaviour to that which we were investigating. ‘Getting a tattoo’ is cited as a disinhibited risk-taking behaviour by^24^ but we have not included it in our classification as there is some historic and ‘outmoded’ stigma associated with tattoos.^87^ suggest that tattoos should not be “viewed as signs of pathology …but opportunities to explore aspects of self-identity that can be valuable in clinical work”.
