# Supplementary material for: Lived experience at the core: A classification system for risk-taking behaviours in bipolar
Source: Digit Health. 2024 Aug 5;10:20552076241269580. doi: 10.1177/20552076241269580 (PMC11301771; doi:10.1177/20552076241269580)
Supplement: sj-docx-1-dhj-10.1177_20552076241269580 - Supplemental material for Lived experience at the core: A classification system for risk-taking behaviours in bipolar [file sj-docx-1-dhj-10.1177_20552076241269580.docx]

Supplementary Materials: A Corpus-Assisted Framework for the Classification of Risk-Taking Behaviours in Bipolar using Lived Experience Interviews

Daisy Harvey^1*^, Paul Rayson^2^, Fiona Lobban^1^, Jasper Palmier-Claus^1,3^, Steven Jones^1^

^1^Spectrum Centre for Mental Health Research, Division of Health Research, Faculty of Health and Medicine, Lancaster University, Lancaster, United Kingdom

^2^UCREL Research Centre, School of Computing and Communications, InfoLab21, Lancaster University, Lancaster, United Kingdom

^3^Lancashire & South Cumbria NHS Foundation Trust, Lancashire, United Kingdom

# Supplementary Data

## Participant Information Sheet (PWLE)

***Building a Risk-Taking Lexicon for Bipolar Disorder***

My name is Daisy Harvey and I am conducting this research as part of my PhD in the department of Health Research at Lancaster University, Lancaster, United Kingdom. I would like to invite you to take part in a study that aims to understand more about the risk-taking behaviours that are talked about by people living with bipolar disorder. This information sheet aims to help you understand the study in more detail and also will also explain your role should you be interested in participating. Please take time to read through this information as it is important that you understand why the research is being completed and what you will be asked to do should you participate. Thank you for taking the time to read this.

**What is the study about?**

The purpose of this study is to use transcribed data from interviews for linguistic analysis, to learn more about the types of risk-taking behaviours that are talked about by people living with bipolar and clinicians who treat individuals with bipolar, and to build a lexicon of risk-taking terminology from this data that will be utilised in later studies within the PhD. It is hoped that this research will provide more information on the types and frequency of risky behaviour which can ultimately be used to guide strategies to improve quality of life and reduce the risk of harm to people living with bipolar.

**Why have I been approached?**

You have been approached because the study requires information from people who have self-reported a diagnosis of bipolar.

**Do I have to take part?**

No, it is completely up to you whether you decide to take part in this study. If you do decide to take part, you will be asked to provide your written or verbal consent, and you may withdraw your consent up to two weeks after the interview has taken place.

**What will I be asked to do if I take part?**

If you decide you would like to take part, you would first be asked to take part in a screening interview to assess your eligibility to take part in the study and confirm a prior diagnosis of bipolar, which should not take longer than 15 minutes. If you are eligible, you will be invited to participate in a one-to-one interview within two weeks of the screening call facilitated by the primary researcher, which will last around 1.5 hours. The interview will include both open-ended questions (~1hr) and a survey style ranking task (~30 minutes). The interview will be arranged at your preferred location, either in person (dependent on COVID-19 restrictions) or remotely via a phone or video call.

**Will my data be identifiable?**

All data collected from the interviews will be made anonymous using pseudonyms or an identification number. Interviews will be recorded using Microsoft Teams or using an iPhone which are both encrypted, and all data collected will be stored securely on Microsoft Onedrive or Microsoft Teams in accordance with Lancaster University’s guidelines. Only the primary researcher, their supervisors and auditors will be granted access to this data. Where it is necessary to store files on a computer hard drive, this data will be encrypted and the computer will be password protected.

Transcription will be performed using NVivo transcription services which meets the university’s criteria for special category data, and which has a GDPR compliant agreement with the university. The recordings of the interviews will be kept until after examination/publication, and then destroyed. The typed version of your interview will be made anonymous by removing any identifying information including your name. Anonymised direct quotations from your interview may be used in the reports or publications from the study, but your name will not be attached to them. All reasonable steps will be taken to protect the anonymity of the participants involved in this project.

A database of text (a corpus) will be generated from all of the transcribed interviews conducted during this study and used to build a risk-taking lexicon. Upon completion of the PhD, the anonymised corpus generated from the interviews will be deposited indefinitely with the UK data service as requested by the funder of this PhD, the ESRC. Due to the sensitivity of this research, the corpus will be deposited as safeguarded data and access will only be granted to researchers with an institutional email address on a case-by-case basis and decided by the directors of the Spectrum Centre and UCREL at Lancaster University. Researchers who request access to the data must agree to an End User Licence which dictates ethical usage of the dataset.

Personal information will be kept separately from the interview transcripts and will be destroyed after 24 months.

**Disclosure Policy**Everything that is spoken about in the interview will remain confidential, unless what is said makes the researcher think that the participant, or someone else, is at significant risk of harm. This means a current risk of harm and will be considered within the context of the interview. Given the sensitive nature of the research which is asking about risky behaviours, discussion of past behaviour or events which could potentially describe historic harm to the participant or others, and where the interviewer assesses that the participant is not describing a present risk to self or others will not normally warrant disclosure.

In the case that the researcher assesses a current risk of harm to self or others, and where possible, the researcher will decide together with the participant the best way to proceed e.g. informing the healthcare provider, and only in a crisis situation will the researcher break confidentiality to seek advice from one of the clinical psychologists in the research team, or contact an emergency service. Where possible, the researcher will inform the participant that they have spoken to an external party.

For further information about how Lancaster University processes personal data for research purposes and your data rights please visit our webpage: [www.lancaster.ac.uk/research/data-protection](http://www.lancaster.ac.uk/research/data-protection)

**What will happen to the results?**

The results will be used to complete a study that forms part of the researcher’s PhD project. This will be included within the thesis for this PhD and may be submitted for publication in an academic or professional journal. If you are interested in receiving a copy of the paper should the final report be published within an academic journal, please inform the researcher who will provide this.

The risk-taking lexicon which will be built using aggregate data from this study will be utilised in future studies as a dictionary search tool on Reddit data and within de-identified medical records.

**Are there any risks?**

The interview will include some questions that ask about personal information, and answering these questions may recall some distressing events. You will be under no obligation to answer any of the questions and can also refuse to answer any of the questions. If the interview causes you distress during or after participation, you are encouraged to inform the researcher and your support network, and contact the resources provided at the end of this sheet.

**Are there any benefits to taking part?**

You will be provided with the choice of a £30 voucher or cash payment for completing the interview with the researcher. You may also find it interesting to discuss your experiences and take part in the research.

**Who has reviewed the project?**

This study has been reviewed and approved by the Faculty of Health and Medicine Research Ethics Committee at Lancaster University (FHMREC21042) and received input from the Spectrum Advisory Panel (a public and patient group). It is funded by the ESRC under grant number ES/P000665/1.

**Where can I obtain further information about the study if I need it?**

If you have any questions about the study, please contact the main researcher:

**Daisy Harvey**

**Email**: [d.harvey4@lancaster.ac.uk](mailto:d.harvey4@lancaster.ac.uk)

Or alternatively one of the supervisors for this PhD:

- **Professor Steve Jones**

**Email:** [s.jones7@lancaster.ac.uk](mailto:s.jones7@lancaster.ac.uk)

- **Professor Paul Rayson**

Email : [p.rayson@lancaster.ac.uk](mailto:p.rayson@lancaster.ac.uk)

- **Professor Fiona Lobban**

Email : [f.lobban@lancaster.ac.uk](mailto:f.lobban@lancaster.ac.uk)

- **Dr Jasper Palmier-Claus**

**Email :** [j.palmier-claus@lancaster.ac.uk](mailto:j.palmier-claus@lancaster.ac.uk)

**Complaints**

If you wish to make a complaint or raise concerns about any aspect of this study and do not want to speak to the researcher, you can contact:

Dr Laura Machin Tel: +44 (0)1524 594973

Chair of FHM REC

Email: l.machin@lancaster.ac.uk

Faculty of Health and Medicine

(Lancaster Medical School)

Lancaster University

Lancaster

LA1 4YG

**Thank you for taking the time to read this information sheet.**

**Resources in the event of distress**

Should you feel distressed either as a result of taking part, or in the future, the following resources may be of assistance.

**NHS 111**

Call 111 for free on a mobile or landline number

Or alternatively, go online to 111.nhs.uk

(Available 24 hours a day, 7 days a week)

**Mind InfoLine**

Phone: 0300 123 3393 (open 9am to 6pm, Monday to Friday)

Email: [info@mind.org.uk](mailto:info@mind.org.uk)

**Samaritans**

Email: [jo@samaritans.org](mailto:jo@samaritans.org)

Phone: 116 123 (available 24 hours a day, 365 days a year)

**SANEline**

Phone: 0300 304 7000 (open every day of the year from 4pm to 10pm)

**Shout 85258**

Text **SHOUT** to 85258 for 24/7 access to a trained Shout volunteer.

**Any emergencies should be directed to the emergency services by calling 999.**

## Participant Information Sheet (HP)

***Building a Risk-Taking Lexicon for Bipolar Disorder***

My name is Daisy Harvey and I am conducting this research as part of my PhD in the department of Health Research at Lancaster University, Lancaster, United Kingdom. I would like to invite you to take part in a study that aims to understand more about the risk-taking behaviours that are talked about by people living with bipolar disorder. This information sheet aims to help you understand the study in more detail and will also explain your role should you be interested in participating. Please take time to read through this information as it is important that you understand why the research is being completed and what you will be asked to do should you participate. Thank you for taking the time to read this.

**What is the study about?**

The purpose of this study is to use transcribed data from interviews for linguistic analysis, to learn more about the types of risk-taking behaviours that are talked about by people living with bipolar and healthcare professionals who work with individuals diagnosed with bipolar, and to build a lexicon of risk-taking terminology from this data that will be utilised in later studies within the PhD. It is hoped that this research will provide more information on the types and frequency of risky behaviour which can ultimately be used to guide strategies to improve quality of life and reduce the risk of harm to people living with bipolar.

**Why have I been approached?**

You have been approached because the study requires information from healthcare professionals who have worked with individuals with bipolar and have 5+ years of experience.

**Do I have to take part?**

No, it is completely up to you whether you decide to take part in this study. If you do decide to take part, you will be asked to provide your written or verbal consent, and you may withdraw your consent up to two weeks after the interview has taken place.

**What will I be asked to do if I take part?**

If you are eligible, you will be invited to participate in a one-to-one interview facilitated by the primary researcher, which will last around 1 hour. The interview will include both open-ended questions and a Likert-item ranking task. The interview will be arranged at your preferred location, either in person (dependent on COVID-19 restrictions) or remotely via a phone or video call.

**Will my data be identifiable?**

All data collected from the interviews will be made anonymous using pseudonyms or an identification number. Interviews will be recorded using Microsoft Teams or an iPhone which are both encrypted, and all data collected will be stored securely on Microsoft Onedrive or Microsoft Teams in accordance with Lancaster University’s guidelines. Only the primary researcher, their supervisors and auditors will be granted access to this data. Where it is necessary to store files on a computer hard drive, this data will be encrypted and the computer will be password protected.

Transcription will be performed using NVivo transcription services which meets the university’s criteria for special category data, and which has a GDPR compliant agreement with the university. The recordings of the interviews will be kept until after examination/publication, and then destroyed. The typed version of your interview will be made anonymous by removing any identifying information including your name. Anonymised direct quotations from your interview may be used in the reports or publications from the study, but your name will not be attached to them. All reasonable steps will be taken to protect the anonymity of the participants involved in this project.

A database of text (a corpus) will be generated from all of the transcribed interviews conducted during this study and used to build a risk-taking lexicon. Upon completion of the PhD, the anonymised corpus generated from the interviews will be deposited indefinitely with the UK data service as requested by the funder of this PhD, the ESRC. Due to the sensitivity of this research, the corpus will be deposited as safeguarded data and access will only be granted to researchers with an institutional email address on a case-by-case basis and decided by the directors of the Spectrum Centre and UCREL at Lancaster University. Researchers who request access to the data must agree to an End User Licence which dictates ethical usage of the dataset.

Personal information will be kept separately from the interview transcripts and will be destroyed after 24 months.

There are some limits to confidentiality: if what is said in the interview makes me think that you, or someone else, is at significant risk of harm, I will have to break confidentiality and speak to a member of staff about this. If possible, I will tell you if I have to do this.

For further information about how Lancaster University processes personal data for research purposes and your data rights please visit our webpage: [www.lancaster.ac.uk/research/data-protection](http://www.lancaster.ac.uk/research/data-protection)

**What will happen to the results?**

The results will be used to complete a study that forms part of the researcher’s PhD project. This will be included within the thesis for this PhD and may be submitted for publication in an academic or professional journal. If you are interested in receiving a copy of the paper should the final report be published within an academic journal, please inform the researcher who will provide this.

The risk-taking lexicon which will be built using aggregate data from this study will be utilised in future studies as a dictionary search tool on Reddit data and within de-identified medical records.

**Are there any risks?**

The interview will include some questions that ask about personal information, and answering these questions may recall some distressing events. You will be under no obligation to answer any of the questions and can also refuse to answer any of the questions. If the interview causes you distress during or after participation, you are encouraged to inform the researcher and your support network, and contact the resources provided at the end of this sheet.

**Are there any benefits to taking part?**

You will be provided with the choice of a £30 voucher, cash payment or donation to a charity of your choice for completing the interview with the researcher. You may also find it interesting to discuss your experiences and take part in the research.

W**ho has reviewed the project?**

This study has been reviewed and approved by the Faculty of Health and Medicine Research Ethics Committee at Lancaster University (FHMREC21042) and received input from the Spectrum Advisory Panel (a public and patient group). It is funded by the ESRC under grant number ES/P000665/1.

**Where can I obtain further information about the study if I need it?**

If you have any questions about the study, please contact the main researcher:

**Daisy Harvey**

**Email**: [d.harvey4@lancaster.ac.uk](mailto:d.harvey4@lancaster.ac.uk)

Or alternatively one of the supervisors for this PhD:

- **Professor Steve Jones**

**Email:** [s.jones7@lancaster.ac.uk](mailto:s.jones7@lancaster.ac.uk)

- **Professor Paul Rayson**

Email : [p.rayson@lancaster.ac.uk](mailto:p.rayson@lancaster.ac.uk)

- **Professor Fiona Lobban**

Email : [f.lobban@lancaster.ac.uk](mailto:f.lobban@lancaster.ac.uk)

- **Dr Jasper Palmier-Claus**

**Email :** [j.palmier-claus@lancaster.ac.uk](mailto:j.palmier-claus@lancaster.ac.uk)

**Complaints**

If you wish to make a complaint or raise concerns about any aspect of this study and do not want to speak to the researcher, you can contact:

Dr Laura Machin Tel: +44 (0)1524 594973

Chair of FHM REC

Email: l.machin@lancaster.ac.uk

Faculty of Health and Medicine

(Lancaster Medical School)

Lancaster University

Lancaster

LA1 4YG

**Thank you for taking the time to read this information sheet.**

**Resources in the event of distress**

Should you feel distressed either as a result of taking part, or in the future, the following resources may be of assistance.

**NHS 111**

Call 111 for free on a mobile or landline number (available 24 hours a day, 7 days a week).

Alternatively, go online to 111.nhs.uk

**Mind InfoLine**

Phone: 0300 123 3393 (open 9am to 6pm, Monday to Friday).

Email: [info@mind.org.uk](mailto:info@mind.org.uk)

**Samaritans**

Email: [jo@samaritans.org](mailto:jo@samaritans.org)

Phone: 116 123 (available 24 hours a day, 365 days a year).

**SANEline**

Phone: 0300 304 7000 (open every day of the year from 4pm to 10pm).

**Shout 85258**

Text **SHOUT** to 85258 for 24/7 access to a trained Shout volunteer.

**Any emergencies should be directed to the emergency services by calling 999.**

## Interview Schedule PWLE

Interview themes (1) Defining risk-taking and personal experiences of risk-taking, (2) The impact of risk-taking behaviours, (3) Feelings and emotions related to risk-taking, and (4) Access to support.

**Introductions and disclosure statement, confirm consent again verbally**

**Opening Questions**

- Can you tell me about yourself e.g., Where you live, what you like to do, any hobbies you have?
- How do you refer to your diagnosis? (e.g., prompts - bipolar, bipolar disorder, manic depressive)
- Can you tell me about when you first diagnosed?
  - What were some of the symptoms you were experience that led to your diagnosis?
  - Can I ask if you take medication for bipolar?

**Part 1 – Main Interview Questions**

**Defining risk-taking**

- How would you define the term risk-taking?
- Can you tell me about any of your risky behaviours?
- Can you tell me about the relationship between mood and risky behaviour X?
- Can you tell me about the relationship between medication and risky behaviours?
- What are the biggest impacts that risky behaviour (X) has had on you or other people?
- What emotions do you associate with this risky behaviour?
  - How does doing something this make you feel at the time?
  - How do you feel about that [risky behaviour] when you reflect back?
- Thank you for sharing that with me. Are there any other risky behaviours that you’d be happy to talk about?
- Are there any risky behaviours that you associate with positive outcomes?

**Support**

- Considering the risky behaviours we've talked about today, are there any that you would like help for?
  - What kind of support would you like?
  - How would you ideally like to access this help?
  - Have you ever tried to seek help for [risky behaviour X]?
    - What did you do?
    - How did that go?
- Do health professionals ever ask you about risky behaviours?
  - Which ones do they ask about?
  - Are there any we have talked about they don’t ask about?

**Part 2 – Examining Risk Taking Behaviours from Literature**

The next part of the interview includes a survey of 17 risk-taking behaviours that have been cited as being relevant to the study of risk-taking in the literature. I’d like you rank each behaviour according to how often you may have engaged with that behaviour (1: Never, 5: Frequently). If there are any behaviours that you don’t feel comfortable talking about, please skip them. I'll give you some time to complete the online form and then I'd like to ask a few follow-up questions.

Qualtrics Link: <https://lancasteruni.eu.qualtrics.com/jfe/form/SV_em7EjGYDfIZRyHY>

Follow up questions:

- Are there any risky behaviours that aren’t included in the questionnaire that you think are important to consider in the future?
- Have you ever been asked to complete a questionnaire which asks about risky behaviours before?

**Closing Questions - Inform participant that I’m turning the recording off.**

**Provide participant with debrief sheet.**

## Interview Schedule HP

Interview themes (1) Defining risk-taking and personal experiences of risk-taking, (2) The impact of risk-taking behaviours, (3) Feelings and emotions related to risk-taking, and (4) Access to support.

**Introductions and disclosure statement, confirm consent again verbally**

**Opening Questions**

- Can you tell me about yourself? e.g., name, age, occupation, where you work
- Can you tell me more about your work?
  - How frequently do you treat people with a bipolar diagnosis?
  - How long have you had experience in this area?
  - In what capacity do you usually work with people who have a diagnosis of bipolar e.g. NHS/ private/ therapy?

**Part 1 – Main Interview Questions**

- How would you define risk-taking?
- From your professional experience, can you tell me a bit about the types of risky behaviour people living with bipolar might engage with?
  - Can you tell me about any other risk-taking experiences?
  - Do service users talk openly about their risk-taking behaviours?
- What are the biggest impacts that you have observed as a result of risky behaviours?
- Do you see any relationship between medication adherence and risky behaviours?
- What are the negative outcomes associated with risk-taking?
  - Are there some positive outcomes associated with risk-taking?
- What type of support is available to service users who may be thinking about taking risks?
  - Do you think that the available support allows people to talk openly about their experiences?

**Part 2 – Examining Risk Taking Behaviours from Literature**

Qualtrics Link: <https://lancasteruni.eu.qualtrics.com/jfe/form/SV_9vHziwKcsoScZFQ>

The next part of the interview includes a survey of 17 risk-taking behaviours that have been cited as being relevant to the study of risk-taking in the literature. I’d like you rank each behaviour according to how often you have observed that behaviour in clients – either by self-report or observation – (1: I have never observed this behaviour, 5: I frequently observe this behaviour). If there are any behaviours that you don’t feel comfortable talking about, please skip them.

- Are there any behaviours that aren’t described in the questionnaire that are important to consider in the future?
- Are you aware of any risk-taking questionnaires that can be used to assess risk-taking with service users?
  - Do you think such a questionnaire would be helpful/ useful?

**Closing Questions - Inform participant that I’m turning the recording off.**

**Provide participant with debrief sheet.**

# Supplementary Figures and Tables

## Suggested Classification System for Risk-Taking Behaviours ^[[1]](#footnote-2)^

| Risky Behaviours and domains | % of total risky behaviours referenced in PWLE corpus (n/N) N = 267 | % of PWLE participants who reference behaviour (n/N) N = 18 | PWLE Mean (mentions of risk-taking behaviour by participant) | PWLE Range (mentions of risking taking behaviour by participant) | PWLE STD (mentions of risking taking behaviour by participant) | % of total risky behaviours referenced in HP corpus (n/N) N = 128 | % of HP participants who reference behaviour (n/N) N =5 | HP Mean (mentions of risk-taking behaviour by participant) | HP Range (mentions of risking taking behaviour by participant) | HP STD (mentions of risking taking behaviour by participant) |
| --- | --- | --- | --- | --- | --- | --- | --- | --- | --- | --- |
| (Anti-) Social behaviours | **16.61% (45/271)** | **83.33% (15/18)** | **7.6** | **11** | **4.03** | **15.62% (20/128)** | **80% (4/5)** | **3.33** | **6** | **2.07** |
| "Having no filter" (being rude/ inappropriate/ lack of social boundaries) | 5.17% (14/271) | 44.44% (8/18) | 1.75 | 6 | 2.12 | 2.34% (3/128) | 20% (1/5) | N/A | N/A | N/A |
| Starting arguments | 2.58% (7/271) | 27.78% (5/18) | 1.4 | 1 | 0.55 | 2.34% (3/128) | 40% (2/5) | 1.5 | 1 | 0.71 |
| Neglecting relationships | 1.48% (4/271) | 22.22% (4/18) | 1 | 0 | 0 | 0.78% (1/128) | 20% (1/5) | N/A | N/A | N/A |
| Hyperfixation | 1.11% (3/271) | 11.11% (2/18) | 1.5 | 1 | 0.71 | 3.13% (4/128) | 40% (2/5) | 2 | 0 | 0 |
| Being aggressive, violent or destructive | 3.69% (10/271) | 38.89% (7/18) | 1.4 | 1 | 0.53 | 5.47% (7/128) | 60% (3/5) | 2.33 | 3 | 1.53 |
| Talking to strangers | 2.58% (7/271) | 22.22% (4/18) | 1.8 | 2 | 0.96 | 1.56% (2/128) | 40% (2/5) | 1 | 0 | 0 |
|  |  | | | | | | | | | |
| Financial behaviours | **13.64% (37/271)** | **77.78% (14/18)** | **6.75** | **19** | **8.47** | **14.85% (19/128)** | **80% (4/5)** | **3.8** | **9** | **3.56** |
| Spending money impulsively or excessively | 7.74% (21/271) | 50% (9/18) | 2.3 | 4 | 1.41 | 7.03% (9/128) | 80% (4/5) | 2.2 | 4 | 1.89 |
| High-risk investing | 0.73% (2/271) | 11.11% (2/18) | 1 | 0 | 0 | Not described narratively by participants during interview but reported in the risk-taking questionnaire. | | | | |
| Gambling | 1.48% (4/271) | 22.22% (4/18) | 1 | 0 | 0 | 3.13% (4/128) | 80% (4/5) | 1 | 0 | 0 |
| Quitting a job impulsively | -- | -- | -- | -- | -- | 3.91% (5/128) | 20% (1/5) | N/A | N/A | N/A |
| Excessive generosity (giving money/belongings away) | 3.69% (10/271) | 44.44% (8/18) | 1.3 | 1 | 0.46 | 0.78% (1/128) | 20% (1/5) | N/A | N/A | N/A |
|  |  | | | | | | | | | |
| Dangerous and disinhibited behaviours | **28.04% (76/271)** | **94% (17/18)** | **7.2** | **18** | **6.2** | **19.53% (25/128)** | **80% (4/5)** | **2.72** | **6** | **2.05** |
| Going to dangerous or unusual places | 4.42% (12/271) | 50% (9/18) | 1.3 | 3 | 1 | 3.91% (5/128) | 60% (3/5) | 1.67 | 2 | 1.15 |
| Leaving home or running away | 3.69% (10/271) | 33.33% (6/18) | 1.7 | 3 | 1.21 | 3.13% (4/128) | 40% (2/5) | 2 | 2 | 1.41 |
| Standing on bridges/ high places | 1.85% (5/271) | 22.22% (4/18) | 1.3 | 1 | 0.5 | 2.34% (3/128) | 40% (2/5) | 1.5 | 1 | 0.71 |
| Impulsive travelling | 0.73% (2/271) | 11.11% (2/18) | 1.0 | 0 | 0 | 1.56% (2/128) | 40% (2/5) | 1 | 0 | 0 |
| Extreme sports | 5.54% (15/271) | 16.67% (3/18) | 5 | 5 | 2.65 | -- | -- | -- | -- | -- |
| Dangerous driving | 7.01% (19/271) | 38.89% (7/18) | 2.7 | 7 | 2.63 | 4.69% (6/128) | 60% (3/5) | 2 | 2 | 1 |
| Stealing | 0.36% (1/271) | 5.56% (1/18) | N/A | N/A | N/A | 1.56% (2/128) | 40% (2/5) | 1 | 0 | 0 |
| Carrying or using weapons | -- | -- | -- | -- | -- | 1.56% (2/128) | 20% (1/5) | N/A | N/A | N/A |
| Wearing inappropriate clothing | 1.11% (3/271) | 11.11% (2/18) | 1.5 | 1 | 0.71 | 0.78% (1/128) | 20% (1/5) | N/A | N/A | N/A |
| Entering vulnerable relationships | 1.85% (5/271) | 11.11% (2/18) | 2.5 | 1 | 0.71 | -- | -- | -- | -- | -- |
| Walking in traffic | 1.48% (4/271) | 22.22% (4/18) | 1 | 0 | 0 | -- | -- | -- | -- | -- |
| Fire starting | Not described narratively by participants during interview but reported in the risk-taking questionnaire. | | | | | | | | | |
|  |  | | | | | | | | | |
| Health-risk and substance-misuse behaviours | **16.23% (44/271)** | **72.22% (13/18)** | **5.6** | **14** | **5.85** | **21.09% (27/128)** | **100% (5/5)** | **3.33** | **5** | **2.52** |
| Medication non-adherence | 4.06% (11/271) | 33.33% (6/18) | 1.8 | 3 | 1.17 | 4.69% (6/128) | 80% (4/5) | 1.5 | 1 | 0.58 |
| Excessive eating | 1.85% (5/271) | 27.78% (5/18) | 1 | 0 | 0 | 2.34% (3/128) | 60% (3/5) | 1 | 0 | 0 |
| Not eating | 0.36% (1/271) | 5.56% (1/18) | N/A | N/A | N/A | 0.78% (1/128) | 20% (1/5) | N/A | N/A | N/A |
| Smoking | 0.36% (1/271) | 5.56% (1/18) | N/A | N/A | N/A | -- | -- | -- | -- | -- |
| Drinking alcohol excessively | 5.54% (15/271) | 38.89% (7/18) | 2.1 | 4 | 1.46 | 7.03% (9/128) | 100% (5/5) | 1.8 | 3 | 1.3 |
| Taking recreational drugs | 4.06% (11/271) | 33.33% (6/18) | 1.8 | 3 | 1.17 | 6.25% (8/128) | 100% (5/5) | 1.6 | 2 | 0.89 |
|  |  | | | | | | | | | |
| Self-injurious and suicidal behaviours | **16.61% (45/271)** | **77.78% (14/18)** | **11.25** | **14** | **6.23** | **20.32% (26/128)** | **100% (5/5)** | **6.5** | **8** | **3.42** |
| Suicide or attempting suicide | 3.69% (10/271) | 27.78% (5/18) | 2 | 2 | 0.71 | 7.82% (10/128) | 80% (4/5) | 2.5 | 2 | 1 |
| Suicidal ideation | 6.27% (17/271) | 50% (9/18) | 1.9 | 2 | 0.78 | 4.69% (6/128) | 60% (3/5) | 2 | 0 | 0 |
| Overdosing | 1.11% (3/271) | 16.67% (3/18) | 1 | 0 | 0 | 1.56% (2/128) | 20% (1/5) | N/A | N/A | N/A |
| Self-harming | 5.54% (15/271) | 44.44% (8/18) | 1.9 | 2 | 0.99 | 6.25% (8/128) | 100% (5/5) | 1.6 | 2 | 0.89 |
|  |  | | | | | | | | | |
| Sexual behaviours | **8.87% (24/271)** | **61.11% (11/18)** | **4** | **7** | **3.02** | **8.59% (11/128)** | **60% (3/5)** | **1.83** | **5** | **2.04** |
| Having sex with strangers | 1.11% (3/271) | 16.67% (3/18) | 1 | 0 | 0 | 0.78% (1/128) | 20% (1/5) | N/A | N/A | N/A |
| Having unprotected sex | 1.85% (5/271) | 22.22% (4/18) | 1.3 | 1 | 0.5 | 0.78% (1/128) | 20% (1/5) | N/A | N/A | N/A |
| Having an affair | 1.11% (3/271) | 16.67% (3/18) | 1 | 0 | 0 | 0.78% (1/128) | 20% (1/5) | N/A | N/A | N/A |
| Porn addiction | -- | -- | -- | -- | -- | 0.78% (1/128) | 20% (1/5) | N/A | N/A | N/A |
| Hypersexuality (dating apps) | 1.11% (3/271) | 16.67% (3/18) | 1 | 0 | 0 | 0.78% (1/128) | 20% (1/5) | N/A | N/A | N/A |
| Hypersexuality (non-specific) | 3.69% (10/271) | 44.44% (8/18) | 1.3 | 1 | 0.46 | 4.69% (6/128) | 60% (3/5) | 2 | 0 | 0 |

*Table 1 A classification of risk-taking behaviours using participant data from qualitative interviews and the risk-taking questionnaire.*

## Additional analysis of risk-taking behaviour mentions by interview task

Figures 1-4 provide the breakdown of behaviour mentions by interview section i.e. Part I - the open-ended interview questions and Part II - the risk-taking questionnaire task; demonstrating how risk-taking behaviour mentions were distributed across the two interview tasks^[[2]](#footnote-3)^. Figure 1 demonstrates that the most frequently mentioned behaviours during the open-ended interview questions for the PWLE group were dangerous driving, spending money excessively, extreme sports, suicidal ideation and self-harming. Figure 2 shows that during Part II of the interviews, the most mentioned behaviours for the same group were excessive eating, spending money excessively, medication non-adherence, talking to strangers and starting arguments. Some of the behaviours discussed during the second part of the interview were influenced by the behaviours included in the risk-taking questionnaire, as participants often provided their answers to the questionnaire accompanied with a narrative description of the risk-taking behaviour. Excessive eating was described by five of the participants during the second part of the interview because it was included as one of the behaviours in the questionnaire but was not mentioned by any of the interview participants before they were provided with this prompt. A number of participants attributed excessive eating to a side effect of their medication, see (1) but other academic research suggests that “eating pathology may occur comorbidly with BD due to shared underlying pathophysiological features” (2). Medication non-adherence was also mentioned by PWLE participants during this section of the interview despite not being included as a risk-taking behaviour within the questionnaire as a number of participants believed that it should be assessed as a risk-taking behaviour in future measurement tools. Figure 6 demonstrates that HP participants only mentioned the risk-taking behaviour of ‘going to dangerous or unusual places’ after viewing this behaviour within the questionnaire, although this was one of the most frequently cited behaviours by PWLE participants.

Figure 2 PWLE group risk-taking behaviour mentions during the risk-taking questionnaire

Figure 1 PWLE group risk-taking behaviour mentions during the open-ended interview questions

Figure 4 HP group risk-taking behaviour mentions during the risk-taking questionnaire

Figure 3 HP group risk-taking behaviour mentions during the open-ended interview questions

## Risk-Taking Behaviour Resources

Table 2 provides a synopsis of the sources which have been used to compile a list of risk-taking behaviours which are currently acknowledged in risk-taking measurement tools or related academic and diagnostic literature. The full charting of these behaviours is located in 2.3, Table 3.

| **Measure** | **Author** | **Description** |
| --- | --- | --- |
| **Modified MIS-CAM**  (Incorporating the ‘dangerous activities subscale’ from the Response Styles to Depression Questionnaire-Revised (RSDQ-R) and the Multi-Impulsivity (MIS) Checklist) | (3–7) | *An adaptation of two existing questionnaires into a Likert item survey asking participants how often they have had ideation or have engaged in 16 risky behaviours over the past seven days.* |
| **Revised and Improved 30-Item Domain-Specific Risk-Taking (DOSPERT) Scale (2006)** (Risk-Taking Section Only) | (8,9) | *A Likert-item survey of 30 items to assess the likelihood of participants engaging in risky behaviours.* |
| **The Risk-Taking Index (RTI)** | (10) | *A Likert item survey which asks participants about their risk-taking behaviours across 6 different contexts.* |
| **The DSM-5** | (11) | *Excerpts of text from the DSM-5 manual which reference risky behaviours within hypomanic, manic and depressive episodes, as well as from the text describing diagnostic features of bipolar disorders.* |
| **Disinhibited Behaviours in a Hypomanic State** | (12) | *A list of behaviours that people with a diagnosis of bipolar engaged with during episodes of hypomania.* |

*Table 2 Existing risk-taking behaviour measures and resources*

## Synthesised list of risk-taking behaviours from existing measures and literature

| List of Risky Behaviours from Existing Measures | Tool | Author | Domain |
| --- | --- | --- | --- |
| Binge eating | Modified MIS-CAM | (3–5) | No Domain Specified |
| Going to a potentially dangerous place | Modified MIS-CAM | (3–5) | No Domain Specified |
| Initiate a relationship with a stranger Starting extensive conversations with strangers in public Tell other people what you really think of them | Modified MIS-CAM DSM-5 Disinhibited behaviours while hypomanic | (3–5) (11) (12) | No Domain Specified Manic/Hypomanic episode Hypomanic episode |
| Shoplifting/ stealing | Modified MIS-CAM | (3–5) | No Domain Specified |
| Hit someone or break something Physically threatening/ assultive Be physically abusive towards others | Modified MIS-CAM DSM-5 Disinhibited behaviours while hypomanic | (3–5) (11) (12) | No Domain Specified Manic/Hypomanic episode Hypomanic episode |
| Provoke an argument Be verbally abusive towards others | Modified MIS-CAM Disinhibited behaviours while hypomanic | (3–5) (12) | No Domain Specified Hypomanic episode |
| Set fire to something | Modified MIS-CAM | (3–5) | No Domain Specified |
| Self-harm | Modified MIS-CAM | (3–5) | No Domain Specified |
| Suicidal ideation Recurrent thoughts of death or suicidal ideation without a specific plan | Modified MIS-CAM DSM-5 | (3–5) (11) | No Domain Specified Major Depressive Episode |
| Suicide attempt Suicide attempt or a plan | Modified MIS-CAM DSM-5 | (3–5) (11) | No Domain Specified Major Depressive Episode OR Manic/Hypomanic episode |
| Spending money Engaging in unrestrained buying sprees Giving away possessions | Modified MIS-CAM DSM-5 DSM-5/ Disinhibited behaviours while hypomanic | (3–5) (11) (11,12) | No Domain Specified Manic/Hypomanic episode Manic/Hypomanic episode Hypomanic episode |
| Taking illegal drugs | Modified MIS-CAM | (3–5) | No Domain Specified |
| Irresistible urge to have sex Sexual indiscretions/ sexual promiscuity Be sexually disinhibited | Modified MIS-CAM DSM-5 Disinhibited behaviours while hypomanic | (3–5) (11) (12) | No Domain Specified Manic/Hypomanic episode Hypomanic episode |
| Drinking alcohol excessively High alcohol consumption Drinking heavily at a social function | Modified MIS-CAM RTI DOSPERT | (3–5) (10) (8,9) | No Domain Specified Health Health/Safety |
| Reckless driving e.g driving fast, driving overtaking, cycling/motorcycling without helmet, driving without seatbelt | Modified MIS-CAM RTI DOSPERT DSM-5 Disinhibited behaviours while hypomanic | (3–5) (10) (8,9) (11) (12) | No Domain Specified Safety Health/Safety Manic/Hypomanic episode Hypomanic episode |
| Smoking | RTI | (10) | Health |
| Poor diet | RTI | (10) | Health |
| Engaging in unprotected sex | DOSPERT | (8,9) | Health/Safety |
| Sunbathing without sunscreen | DOSPERT | (8,9) | Health/Safety |
| Walking home alone at night in an unsafe area of town. | DOSPERT | (8,9) | Health/Safety |
| Gambling money Gambling Betting a day's income at the horse races Betting a day’s income at a high-stake poker game Betting a day’s income on the outcome of a sporting event  Gambling and antisocial behaviour | Modified MIS-CAM RTI DOSPERT DOSPERT DOSPERT DSM-5 | (3–5) (10) (8,9) (8,9) (8,9) (11) | No Domain Specified Financial Financial - Gambling Financial - Gambling Financial - Gambling Manic/Hypomanic episode |
| Risky financial investments (stocks/real estate) Risky investments Investing 10% of your annual income in a moderate growth diversified fund Investing 5% of your annual income in a very speculative stock Investing 10% of your annual income in a new business venture Foolish business investments/ serious financial difficulties | Modified MIS-CAM RTI DOSPERT DOSPERT DOSPERT DSM-5 | (3–5) (10) (8,9) (8,9) (8,9) (11) | No Domain Specified Financial Financial - Investment Financial - Investment Financial - Investment Manic/Hypomanic episode |
| Rock climbing  Scuba diving Going whitewater rafting at high water in the spring Going down a ski run that is beyond your ability Going camping in the wildnerness Taking a skydiving class Bungee jumping off a tall bridge Piloting a small plane | RTI RTI DOSPERT DOSPERT DOSPERT DOSPERT DOSPERT DOSPERT | (10) (10) (8,9) (8,9) (8,9) (8,9) (8,9) (8,9) | Recreational Recreational Recreational Recreational Recreational Recreational Recreational Recreational |
| Leaving your young children alone at home while running an errand. | DOSPERT | (8,9) | Ethical |
| Not returning a wallet you found that contains $200 | DOSPERT | (8,9) | Ethical |
| Taking some questionable deductions on your income tax return | DOSPERT | (8,9) | Ethical |
| Having an affair with a married man/woman | DOSPERT | (8,9) | Ethical |
| Passing off somebody else’s work as your own | DOSPERT | (8,9) | Ethical |
| Revealing a friend’s secret to someone else | DOSPERT | (8,9) | Ethical |
| Standing for election | RTI | (10) | Social |
| Publicly challenging a rule or decision Disagreeing with an authority figure on a major issue Speaking your mind about an unpopular issue in a meeting at work Write excessive letters, e-mails, text messages, and so forth, on many different topics to friends, public figures, or the media | RTI DOSPERT DOSPERT DSM-5 | (10) (8,9) (8,9) (11) | Social Social Social Manic/Hypomanic episode |
| Admitting that your tastes are different from those of a friend | DOSPERT | (8,9) | Social |
| Moving to a city far away from your extended family Travel overseas | DOSPERT Disinhibited behaviours while hypomanic | (8,9) (12) | Social Hypomanic episode |
| Choosing a career that you truly enjoy over a more secure one Starting a new career in your mid-thirties Quit a job without another to go to | DOSPERT DOSPERT RTI/ Disinhibited behaviours while hypomanic | (8,9) (8,9) (10,12) | Social Social Career Hypomanic episode |
| Getting a tattoo | Disinhibited behaviours while hypomanic | (12) | Hypomanic episode |

*Table 3 Synthesised list of risk-taking behaviours from existing measures and literature*

Bibliography

1. Theisen FM, Linden A, König IR, Martin M, Remschmidt H, Hebebrand J. Spectrum of binge eating symptomatology in patients treated with clozapine and olanzapine. J Neural Transm [Internet]. 2003;110(1):111–21. Available from: https://doi.org/10.1007/s00702-002-0792-6

2. McDonald CE, Rossell SL, Phillipou A. The comorbidity of eating disorders in bipolar disorder and associated clinical correlates characterised by emotion dysregulation and impulsivity: A systematic review. J Affect Disord [Internet]. 2019;259:228–43. Available from: https://www.sciencedirect.com/science/article/pii/S0165032719309498

3. Reinharth J, Braga R, Serper M. Characterization of risk-taking in adults with bipolar spectrum disorders. J Nerv Ment Dis. 2017;205(7):580–4.

4. Knowles R, Tai S, Christensen I, Bentall R. Coping with depression and vulnerability to mania: A factor analytic study of the Nolen-Hoeksema (1991) Response Styles Questionnaire. British Journal of Clinical Psychology [Internet]. 2005;44(1):99–112. Available from: https://bpspsychub.onlinelibrary.wiley.com/doi/abs/10.1348/014466504X20062

5. Evans CDH, Searle Y, Dolan BM. Two new tools for the assessment of multi-impulsivity: the ‘MIS’and the ‘CAM.’ European Eating Disorders Review: The Professional Journal of the Eating Disorders Association. 1998;6(1):48–57.

6. Nolen-Hoeksema S. Responses to depression and their effects on the duration of depressive episodes. J Abnorm Psychol. 1991;100(4):569.

7. Thomas J, Bentall RP. Hypomanic traits and response styles to depression. British Journal of Clinical Psychology. 2002;41(3):309–13.

8. Weber EU, Blais A, Betz NE. A domain‐specific risk‐attitude scale: Measuring risk perceptions and risk behaviors. J Behav Decis Mak. 2002;15(4):263–90.

9. Blais AR, Weber EU. A domain-specific risk-taking (DOSPERT) scale for adult populations. Judgm Decis Mak. 2006;1(1):33–47.

10. Nicholson N, Soane E, Fenton‐O’Creevy M, Willman P. Personality and domain‐specific risk taking. J Risk Res. 2005;8(2):157–76.

11. American Psychiatric Association. DSM 5 Diagnostic and Statistical Manual of Mental Disorders (5th ed.). American Psychiatric Association. 2013;

12. Fletcher K, Parker G, Paterson A, Synnott H. High-risk behaviour in hypomanic states. J Affect Disord [Internet]. 2013;150(1):50–6. Available from: https://www.sciencedirect.com/science/article/pii/S0165032713001778

1. N/A denotes where the data was provided by only one participant and therefore the mean/ range and standard deviation is not available. [↑](#footnote-ref-2)
2. The graphs which demonstrate risk-taking behaviour mentions during the questionnaire task only include narrative descriptions of risk-taking behaviours and not the answers which participants provided to the questionnaire itself, which are provided in section 3.2.2 of the main paper. [↑](#footnote-ref-3)
