# Supplementary material for: Lived experience at the core: A classification system for risk-taking behaviours in bipolar
Source: Digit Health. 2024 Aug 5;10:20552076241269580. doi: 10.1177/20552076241269580 (PMC11301771; doi:10.1177/20552076241269580)
Supplement: sj-docx-2-dhj-10.1177_20552076241269580 - Supplemental material for Lived experience at the core: A classification system for risk-taking behaviours in bipolar [file sj-docx-2-dhj-10.1177_20552076241269580.docx]

**Consolidated criteria for reporting qualitative studies (COREQ): 32-item checklist for manuscript in title:**

“***Lived Experience at the Core: A Classification System for Risk-Taking Behaviours in Bipolar* ”**

| **No** | **Item** | **Guide questions/description** | **Response** |
| --- | --- | --- | --- |
| **Domain 1: Research team and reflexivity** | | |  |
| Personal Characteristics | | |  |
| 1. | Interviewer/facilitator | Which author/s conducted the interview or focus group? | *Page 6* |
| 2. | Credentials | What were the researcher's credentials? *E.g. PhD, MD* | *Page 6* |
| 3. | Occupation | What was their occupation at the time of the study? | *Page 6* |
| 4. | Gender | Was the researcher male or female? | *Page 6* |
| 5. | Experience and training | What experience or training did the researcher have? | *Page 6* |
| Relationship with participants | | |  |
| 6. | Relationship established | Was a relationship established prior to study commencement? | No |
| 7. | Participant knowledge of the interviewer | What did the participants know about the researcher? e*.g. personal goals, reasons for doing the research* | *See supplementary materials (1.1).*  At the beginning of each interview, we explained the individual goals and reasons for conducting the research. All participants were also provided with an information sheet prior to taking part in the interviews to explain the research rationale. The participant information sheet is provided with the supplementary materials. |
| 8. | Interviewer characteristics | What characteristics were reported about the interviewer/facilitator? e.g. *Bias, assumptions, reasons and interests in the research topic* | *See supplementary materials (1.1).*  The interviewer tried to avoid reporting their own assumptions to avoid influencing participants. However, the participation information sheet provided in the supplementary materials demonstrates the rationale for the research and the importance of this topic. |
| **Domain 2: study design** | | |  |
| Theoretical framework | | |  |
| 9. | Methodological orientation and Theory | What methodological orientation was stated to underpin the study? *e.g. grounded theory, discourse analysis, ethnography, phenomenology, content analysis* | *Page 7*  Grounded theory content analysis. |
| Participant selection | | |  |
| 10. | Sampling | How were participants selected? *e.g. purposive, convenience, consecutive, snowball* | *Page 4*  Purposive sampling. |
| 11. | Method of approach | How were participants approached? e*.g. face-to-face, telephone, mail, email* | *Page 4*  Recruitment advert on ‘People in Research’, on Lancaster University Spectrum Connect or via direct email. |
| 12. | Sample size | How many participants were in the study? | *Page 1 (abstract)*  N = 18 (People with Lived Experience)  N = 5 (Healthcare Professionals) |
| 13. | Non-participation | How many people refused to participate or dropped out? Reasons? | *Page 10 (figure 2)*  18 people with lived experience either dropped out or did not respond to further emails regarding the project.  1 healthcare professional was ultimately removed from the study due to not meeting the inclusion criteria. |
| Setting | | |  |
| 14. | Setting of data collection | Where was the data collected? e*.g. home, clinic, workplace* | *Page 6*  Remote workplace via phone call or Microsoft Teams. |
| 15. | Presence of non-participants | Was anyone else present besides the participants and researchers? | *Page 6*  No-one else was present besides the interviewer and the participant, and all interviews took place remotely with the interviewer working in a private location. |
| 16. | Description of sample | What are the important characteristics of the sample? *e.g. demographic data, date* | *Page 10*  Participant demographics are reported in Table 1. |
| Data collection | | |  |
| 17. | Interview guide | Were questions, prompts, guides provided by the authors? Was it pilot tested? | *Page 5 and 6*  Interview schedules were used to guide the interviews. These are provided in the supplementary materials (1.2 and 1.3). The interview schedule was also pilot tested with on member of the research team and one member of the Lancaster University Spectrum Conenct Advisory Panel who has lived experience. |
| 18. | Repeat interviews | Were repeat interviews carried out? If yes, how many? | *Page 6*  Participants who didn’t satisfy the criteria for the screening questionnaires were invited to complete the screening again two weeks later. No repeat interviews were conducted. |
| 19. | Audio/visual recording | Did the research use audio or visual recording to collect the data? | *Page 5* |
| 20. | Field notes | Were field notes made during and/or after the interview or focus group? | *Page 5*  The interviews were recorded so notes were not taken during the interview to allow the interviewer to be fully present. Transcripts were produced from the interview recordings. |
| 21. | Duration | What was the duration of the interviews or focus group? | *Page 10*  The interviews ranged from 20 to 80 minutes and the average interview length was 45 minutes. |
| 22. | Data saturation | Was data saturation discussed? | *Page 12* |
| 23. | Transcripts returned | Were transcripts returned to participants for comment and/or correction? | No |
| **Domain 3: analysis and findings** | | | |
| Data analysis | | | |
| 24. | Number of data coders | How many data coders coded the data? | *Page 7-8*  DH annotated all of the interview transcripts for risk-taking behaviours, PR independently annotated 40% of the transcripts to calculate agreement. |
| 25. | Description of the coding tree | Did authors provide a description of the coding tree? | *See supplementary materials (2.1).*  The encoded risk-taking behaviours are provided in the supplementary materials (2.1) |
| 26. | Derivation of themes | Were themes identified in advance or derived from the data? | *Method described in 2.3.1, page 7 and 8* |
| 27. | Software | What software, if applicable, was used to manage the data? | NVivo Transcription and Sketch Engine |
| 28. | Participant checking | Did participants provide feedback on the findings? | No |
| **Reporting** | | | |
| 29. | Quotations presented | Were participant quotations presented to illustrate the themes / findings? Was each quotation identified? e*.g. participant number* | *Page 18-22* |
| 30. | Data and findings consistent | Was there consistency between the data presented and the findings? | *Page 22-25*  Described in discussion |
| 31. | Clarity of major themes | Were major themes clearly presented in the findings? | Presented throughout the paper in the classification system and the resulting analyses. |
| 32. | Clarity of minor themes | Is there a description of diverse cases or discussion of minor themes? | *Page 17*  We have included discussion on positive risk-taking although this was not the main focus of the study. |
